# Stabilization of Ni-containing Keggin-type polyoxometalates with variable oxidation states as novel catalysts for electrochemical water oxidation†

**DOI:** 10.1039/d4sc01087f

**Published:** 2024-05-13

**Authors:** Xiang Li, Bryan Kit Yue Ng, Ping-Luen Ho, Chunbo Jia, Jining Shang, Tatchamapan Yoskamtorn, Xuelei Pan, Yiyang Li, Guangchao Li, Tai-Sing Wu, Yun-Liang Soo, Heyong He, Bin Yue, Shik Chi Edman Tsang

**Affiliations:** a Department of Chemistry, University of Oxford Oxford OX1 3QR UK edman.tsang@chem.ox.ac.uk; b Department of Chemistry and Shanghai Key Laboratory of Molecular Catalysis and Innovative Materials, Fudan University Shanghai 200438 China heyonghe@fudan.edu.cn yuebin@fudan.edu.cn; c National Synchrotron Radiation Research Center 101 Hsin-Ann Road Hsinchu 30076 Taiwan

## Abstract

The development of new recyclable and inexpensive electrochemically active species for water oxidation catalysis is the most crucial step for future utilization of renewables. Particularly, transition metal complexes containing internal multiple, cooperative metal centers to couple with redox catalysts in the inorganic Keggin-type polyoxometalate (POM) framework at high potential or under extreme pH conditions would be promising candidates. However, most reported Ni-containing POMs have been highly unstable towards hydrolytic decomposition, which precludes them from application as water oxidation catalysts (WOCs). Here, we have prepared new tri-Ni-containing POMs with variable oxidation states by charge tailored synthetic strategies for the first time and developed them as recyclable POMs for water oxidation catalysts. In addition, by implanting corresponding POM anions into the positively charged MIL-101(Cr) metal–organic framework (MOF), the entrapped Ni^2+^/Ni^3+^ species can show complete recyclability for water oxidation catalysis without encountering uncontrolled hydrolysis of the POM framework. As a result, a low onset potential of approximately 1.46 V *vs.* NHE for water oxidation with stable WOC performance is recorded. Based on this study, rational design and stabilization of other POM-electrocatalysts containing different multiple transition metal centres could be made possible.

## Abstract 2

Stabilization of PW_9_Ni_3_ polyoxometalate (POM) anions in the MIL-101(Cr) framework, which displays high water oxidation activity under electrochemical conditions with the POM remaining intact.

## Graphical abstract

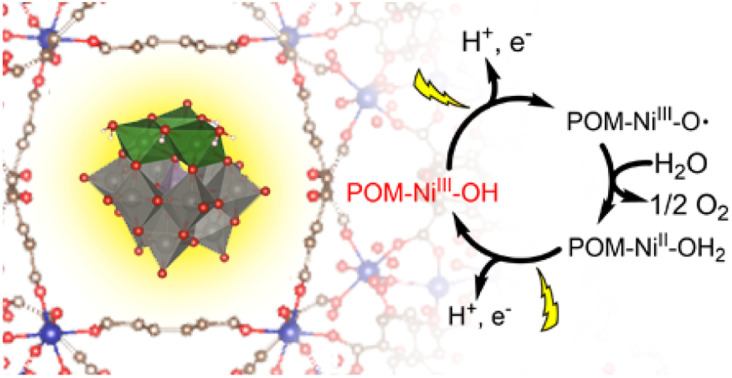

## Introduction

The demand for developing green routes to renewable energy sources and fine chemicals has highlighted the application of polyoxometalate (POM) compounds as redox catalysts due to their outstanding performance in various catalytic oxidations,^1–3^ ability to possess advantages for both homogeneous and heterogeneous catalysis^4^ and high structural diversity and great potential for rational molecular design.^5^ Specifically, multiple transition metal centres or clusters in the POM framework as catalysts without being oxidised at high potential or under extreme pH conditions generate considerable interest in electrochemical water oxidation reactions,^2^ which could form an important part of hydrogen production through the overall electrochemical water splitting process. In the past few decades, the development and evaluation of POM-based electrochemical water oxidation catalysts (WOCs) have proven to be effective under extreme conditions in which most organic ligands or oxide-based WOCs do not have the required long-term stability.^4,6–8^ It is believed that the superior catalytic activity of the substituted POM species originates from their unique electronic structures and fast intra-electron transfer and variable oxidation states of the multiple transition metal centres, in which unusual high-valent states may exist as key intermediates for the oxidation.^9,10^

Typically, some substituent transition metals incorporated into the frameworks of polyoxometalates as the potential active centres of the POM-based electrochemical WOCs, such as ruthenium,^11^ cobalt^4^ and copper^12^ have been reported. The most well-known POM-based WOC reported by Yin *et al.* was [Co_4_(H_2_O)_2_(PW_9_O_34_)_2_]^10−^,^13^ whose chemical properties and stability have extensively been studied in detail. The catalytic mechanism of this Co-based POM WOC was also investigated, in which a Co^3+^-containing {Co^III^–O˙}radical species was believed to be the key species in the catalytic cycle.^9^ Another interesting example would be [Co_9_(H_2_O)_6_(OH)_3_(HPO_4_)_2_(PW_9_O_34_)_3_]^16−^ first developed by Goberna-Ferron *et al.* as a POM-based electrochemical WOC, which displayed good WOC activity under acidic conditions^14,15^ and was able to be heterogenized by precipitation using Cs^+^ as the counter cation.^4^

Despite identifying such potential POM-based WOCs, the pressing issue of their hydrolytic instability remains outstanding, which significantly affects the full evaluation of WOC activities of these molecular catalysts. The potential hydrolysis could release active metal components as corresponding oxide particles which could display an unignorable or even dominant contribution to the WOC activity. Stracke *et al.* emphasised the importance of evaluating “truly” WOC species in cobalt-based POM WOC systems since these unstable catalysts could be rapidly hydrolysed to CoO_*x*_ that contributed significantly towards residue activity,^16^ while studies by Vickers *et al.*, in contrast, pointed out the complexity of the origin of the observed WOC activities.^17^ These comprehensive studies recall the importance of evaluating initial activity and long-term catalytic stability when designing Co-based POM WOCs, which has also brought to attention other multiple metal substituted POMs in WOC.^16^

Nickel is a transition metal element that is widely used in the development of WOCs, especially in the case of relatively stable oxide and hydroxide-based WOCs.^18,19^ Several POM clusters containing multi-Ni centers without vigorous structural proof have been claimed to be active for photocatalytic WOC activity,^20–22^ but the initial ‘genuine’ activities, stability and degrees of contribution from hydrolyzed contaminants of the Ni-based photocatalytic WOCs are questionable.^16^ Given the wide potential application of inexpensive and well-studied Ni-based WOCs in electrochemical water oxidation catalysis, one may anticipate the outstanding electrochemical WOC performance of the multiple nickel centers in POM frameworks as well, if they can be stabilized. However, the present hydrolytic instabilities of Ni-based POMs^23^ have put a critical obstacle on their evaluations, which have prevented Ni-based POMs from being used as electrochemical WOCs. Thus, a realistic way to synthesize, test and stabilize the Ni-based POMs for electrochemical WOC application is urgently required. To understand the origin of instability and achieve stabilization, it's necessary not only to consider their stability prior to reaction but also the genuine catalytic species in the catalytic redox cycle.^24^ In the case of Ni-containing WOCs, high-valent nickel centers such as Ni^3+^ and Ni^4+^ have been considered to be the active intermediates in the catalytic cycle.^25^ Single site Ni^4+^-containing POM compounds have also been reported previously,^26,27^ but the chemistry of Ni^3+^ in multiple Ni metal-POM frameworks remains to be studied. To the best of our knowledge, detailed structural studies for such compounds have not yet been reported. In contrast, previous studies on similar substituted POM WOCs have revealed significant hydrolytic instability of the POM anions with Co^2+^ and Co^3+^.^28^

Herein, we present a detailed comprehensive study including structure–activity relationships, hydrolysis and stabilization of a new redox pair of tri Ni-substituted POMs with different oxidation states, namely [PW_9_Ni_2_^III^Ni^II^(OH)_4_(OH_2_)_2_]^5−^ (1) and [PW_9_Ni_3_^II^(OH)_3_(OH_2_)_3_]^6−^ (2) as potential electrochemical POM WOCs, regarding their physiochemical properties under electrochemical water oxidation reaction conditions. Through extensive investigation of these Ni-based POM catalysts under electrochemical conditions, we have gained a deeper understanding of the intrinsic activity and stability. Ultimately, through the entrapment of the {PW_9_M_3_}-type structure as an example from the class of multi-Ni-substituted polyoxometalates into a MIL-101(Cr) MOF, the electrochemical WOC application of the Ni-based POMs is hereby investigated, which demonstrates the unprecedented outstanding activity and stability as WOCs.

## Experimental section

### Synthesis of compounds Cs_4_K-1 and Cs_4_KH-2

The polyoxometalate Na_8_H[A-α-PW_9_O_34_]·13H_2_O was synthesised according to the literature method and confirmed by IR spectroscopy.^29^ All other reagents used in the synthesis were purchased from Sigma Aldrich. Typically, 500 mg (1.7 mmol) of nickel(ii) nitrate hexahydrate and 2500 mg (0.9 mmol) of Na_8_H[PW_9_O_34_]·13H_2_O were completely dissolved in 15 mL of deionised water in a 50 mL beaker, and the resulting solution was heated to 70 °C in a water bath accompanied by continuous stirring. A solid mixture of 25 mg (0.1 mmol) of silver acetate and 1.65 g (6.1 mmol) of potassium peroxydisulfate was then added to the solution, and the solution was kept at 70 °C with stirring until it became brownish black. The beaker was then cooled down to room temperature in cold water with continuous stirring for a further 10 min. 2 g (10.2 mmol) of caesium nitrate was then added to precipitate the anion 1 formed in the reaction and the mixture was stirred for further 25 min. The brownish-black raw product was then isolated from the suspension by centrifugation and was redissolved in 25 mL of deionised water and heated to 45–55 °C. The solution was then left without stirring at this temperature for sedimentation, and the supernatant was then transferred to a clean glass vial and kept at room temperature overnight. A deep brown precipitate of Cs_4_K-1 appeared and was obtained by centrifugation, dried in air and stored at 0 °C. Typical yield: 340 mg (12% based on W). Spherical aggregates of Cs_4_K-1 can be obtained by slow evaporation of the recrystallised solution at room temperature. Elemental analysis (wt%) result: calcd: W, 51.89; Ni, 5.52; Cs, 16.67; K, 1.23. Found: W, 51.98; Ni, 5.85; Cs, 17.11; K, 1.72.

Compound Cs_4_KH-2 could be obtained by leaving the Cs4K-1 powders and spherical aggregates at room temperature for 2 months. Elemental analysis (wt%) result: calcd: W, 52.15; Ni, 5.55; Cs, 16.76; K, 1.23. Found: W, 52.70; Ni, 5.66; Cs, 17.21; K, 1.69.

### Synthesis of the PW_9_Ni_3_/MIL-101(Cr) composite

The metal–organic framework MIL-101(Cr) was synthesised based on the literature method.^30^ For the synthesis of the composite, typically, 140 mg (0.2 mmol) of MIL-101(Cr) was suspended in 20 mL of deionised water, followed by the addition of 200 mg (0.06 mmol) of Cs_4_K-1 into the mixture. The reaction mixture was sonicated for 20 minutes and was then stirred at room temperature for 24 h. The composite with a yield of 160 mg was isolated from the mixture by centrifugation, washed thoroughly and dried in air.

### General characterisation methods

The ICP-MS elemental analysis was performed with an Agilent 7800 ICP-MS instrument for the amount of W, Ni and K in the samples. The samples were microwave acid digested prior to the analysis and the data are the average value from 3 time measurements. The amount of Cs is determined based on XPS analysis. The IR spectra of compounds were recorded by using a Nicolet iS50 FT-IR spectrometer with a Smart Golden Gate Accessory with ZnSe lenses for diffuse reflectance measurements. The UV-Vis spectra were recorded by using a Shimadzu UV-2600 UV-Vis spectrometer. The concentration of the solution used for the measurement is 1.89 mmol L^−1^ (for the measurement in the range of 500–1200 nm) and 42.0 μmol L^−1^ (for the measurement in the range of 200–500 nm), respectively. The magnetic measurement was conducted on a Quantum Design MPMS-3 SQUID magnetometer. Magnetisation measurement was conducted at 2 K with a variable external magnetic field. Thermogravimetric experiments were performed using a TA instruments Q600 SDT thermogravimetric analyser under an air atmosphere (flow rate: 100.0 mL min^−1^, heating rate: 10.00 °C min^−1^) in the temperature range of 30–700 °C with the starting mass of 7.9676 mg for Cs_4_K-1 and 9.5143 mg for Cs_4_KH-2. Powder X-ray diffraction (PXRD) patterns were recorded by using a Bruker D8 Advance diffractometer. See the ESI† for detailed info on specific characterisation experiments.

### Computational details

All computational studies were conducted using the Gaussian 16 A.03 computational software package^31^ by density functional theory (DFT) methods using the PBE0 hybrid functional^32^ with DFT-D3(Becke-Johnson) dispersion correction.^33,34^ The IEFPCM solvation model was applied to all calculations with water as the solvent to model the solvation effect. Geometric optimisation and simulation of IR spectra of different structures were performed using the def2svp basis set.^35^ All structures are optimised to a local minimum without imaginary frequencies. Optimisation and IR simulation with 6-31G(d)/lanl2dz basis sets were also performed for comparison.^36–40^ In this case, the 6-31G(d) basis set was used for O, P and H atoms while the lanl2dz basis set was applied to all metal atoms. All simulated IR spectra were corrected using precomputed scaling factors. The scaling factors used were 0.9547 for the PBE0-D3BJ/def2svp computational level^41^ and 0.9614 for the PBE0-D3BJ/6-31G(d)/lanl2dz computational level.^42^ Visualization of all optimised structure models was performed using the VESTA 3.5.8 software package.^43^

Single point energies of structures were computed using the def2tzvp basis set. The Gibbs free energy values at 298 K were calculated based on the thermal correction obtained at the PBE0-D3BJ/def2svp computational level and single point energies at the PBE0-D3BJ/def2tzvp level. Prediction of UV-Vis spectra of the anions was performed using the time-dependent density functional theory (TD-DFT) method at the PBE0-D3BJ/def2tzvp computational level. The electron–hole analysis was conducted using the Multiwfn 3.8 software package.^44,45^

### Electrochemical measurements

Electrochemical measurements were performed in 0.2 M K_2_SO_4_ solution with a standard three-electrode electrochemical system on an Ivium VERTEX electrochemical workstation. An Ag/AgCl (saturated KCl) electrode was used as the reference electrode and a Pt wire electrode was used as the counter electrode. The pH values of the corresponding solution were in the range of 7.01–7.13 prior to the tests, and no significant pH change was observed after all experiments illustrated below.

For the measurements of homogeneous systems, a fixed amount of solutes was directly dissolved in the electrolyte prior to the measurement (20 mg for Cs_4_K-1). A glassy carbon electrode (*S*_eff_ = 0.07 cm^2^) was used as the working electrode.

For the heterogeneous measurement of the composite, a typical procedure for the preparation is as follows. 25 mg of the composite was suspended in a mixture of 1.2 mL ethanol, 3.8 mL deionised water and 100 μl of 5 wt% Nafion® ethanol solution. The mixture was sonicated at 30 °C for 20 min, and 300 μl of the resulting suspension was evenly dropped on a 2 × 2 cm carbon paper, followed by drying naturally in air. The heterogeneous measurement of MIL-101(Cr) followed the same procedure, but 25 mg of MIL-101(Cr) instead of the composite was used.

The cyclic voltammetric test was conducted for different potential ranges with a scan rate of 50 mV s^−1^. The linear sweep voltammetric test was performed in the potential range of 0.5–2.0 V *vs.* NHE with a scan rate of 10 mV s^−1^. The differential pulse voltammetric tests were performed for different potential ranges with a pulse time of 10 ms, pulse amplitude of 25 mV, step width of 5 mV, equilibration time of 5 s and scan rate of 50 mV s^−1^. Electrochemical impedance spectroscopy (EIS) measurements were conducted in the frequency range of 0.1 Hz to 100 kHz with an AC voltage of 10 mV amplitude. Data fitting was conducted using the EIS Spectrum Analyser software package.^46^

For durability tests, chronoamperometric measurements were conducted at the applied potential of 1.85 V *vs.* NHE for 5500 s (MIL-101(Cr) and the composite) or 2000 s (homogeneous solution of 1). The extended OER test was performed at 1.85 V *vs.* NHE for 10 000 s.

### Synchrotron X-ray characterization

X-ray absorption spectroscopy (XAS) data of Cs_4_K-1 and Cs_4_KH-2 were measured at beamline TLS07A of Taiwan Light Source at the National Synchrotron Radiation Research Centre (NSRRC). *In situ* XAS data of the PW_9_Ni_3_/MIL-101(Cr) composite were measured at beamline BL11B of Shanghai Synchrotron Radiation Facility (SSRF). Fluorescence mode was used for Ni K-edge measurements and achieved by using a silicon drift detector. To ascertain the reproducibility of the experimental data, at least 3 scan sets were collected and compared for each sample.

The EXAFS data analysis was performed using IFEFFIT with Horae packages (Athena and Artemis). The spectra were calibrated with Ni metal foil as a reference to avoid energy shifts in the samples. And the amplitude reducing parameter was obtained from EXAFS data analysis of the Ni foil, which was used as a fixed input parameter in the data fitting to allow the refinement in the coordination number of the absorption element. In this work, the analysis of the data was performed with the assumption of single scattering with the errors estimated using the R-factor. The data fitting is performed in the R-space with *k*^3^-weighted data along with the background. The K-range of the data fitting is 2–13 Å^−1^ for Cs_4_K-1 and Cs_4_KH-2 and 2–12 Å^−1^ for the PW_9_Ni_3_/MIL-101(Cr) composite (see Fig. 2b, d and S25b†). The R-range of data fitting is 1–4 Å.

Total X-ray scattering was measured at high-energy XRD beamline BL08W of Super Photon ring-8 GeV (SPring-8) synchrotron radiation facility in Japan. The samples were placed in borosilicate capillaries with a diameter of 1.0 mm i.d. and measured using X-rays with a wavelength of 0.1086 Å (*λ* = 115 keV) equipped with a PerkinElmer XRD 1621 CN3 flat-panel detector. Scattering data of standard silicon samples and empty capillaries were also collected for detector correction and background correction purposes, respectively. Pair distribution function (PDF) analysis of the scattering data was conducted using the DAWN software package.^47^ The simulated PDF results of 1 and 2 in different structures were obtained using the xPDFsuite software package^48^ based on optimised structures obtained from computational studies.

## Results and discussion

### Construction and identification of tri-Ni POMs, 1 and 2

Our investigation on the properties of Ni-containing POM WOCs started from direct construction and characterisation of the high-valent Ni^3+^ centres stabilised in POM frameworks. Direct oxidation methods by adding oxidants such as potassium peroxydisulfate into the mixture containing lacunary POM ligands and Ni^2+^ did not lead to any observable desired products. Thus, we then applied the previously developed silver-assisted peroxydisulfate oxidation methods^49^ which can significantly enhance the oxidation ability of the peroxydisulfate. The addition of silver salt catalysts into the reaction mixture led to immediate blackening of the solution, from which a brownish-black solid product can be obtained by the addition of an excess amount of caesium nitrate. Upon further recrystallisation, fine brownish black powder of Cs_4_K[PW_9_Ni_3_(OH)_4_(OH_2_)_2_]·6H_2_O (Cs_4_K-1) containing Ni^3+^ was precipitated from the solution. Large spherical aggregates of Cs_4_K-1 can also be obtained by slow evaporation of the mother liquor.

**Fig. 1:**
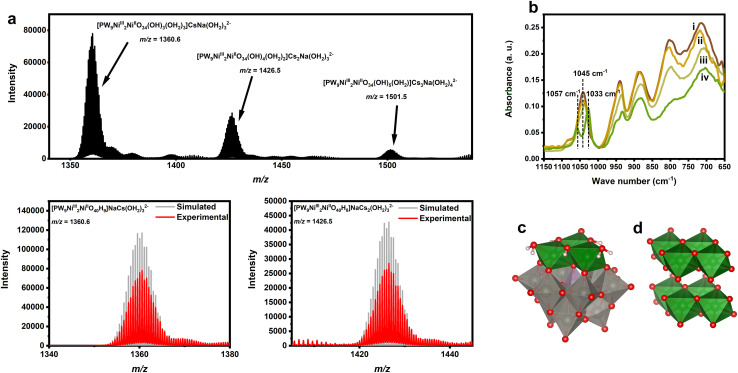
(a) ESI mass spectrum of aqueous solution of Cs_4_K-1. Simulation for peaks at *m*/*z* = 1360.6 and 1426.5 is shown for comparison. (b) IR spectrum of the freshly prepared (i) and partially reduced (ii and iii) Cs_4_K-1 sample and reduced Cs_4_KH-2 sample (iv). (c) Polyhedral structural representation of the computed most stable isomer B-α-1. (d) The crystal structure of Ni(OH)_2_.^50^

The ESI-MS result of Cs_4_K-1 (Fig. 1a) shows three very strong doubly charged peaks attributed to the combination of water molecules, counter cations and POM anions, which seemed to fit [PW_9_Ni_2_^III^Ni^II^O_40_H_*x*_]^(13−*x*)−^ containing two Ni^3+^ centres and one Ni^2+^ centre in each anion, with different protonated states. It should be noted that a small amount of Na^+^ ions were also involved, which did not occur in the compound, but originated from the glass material in the ESI-MS apparatus. No other noticeable peaks are observed, indicating the existence of isolated trisubstituted ions as the only main anionic component in the solution of Cs_4_K-1, without aggregation or fragmentation. The mass spectroscopy peaks can be perfectly simulated assuming that two of three nickel centres display the +3 oxidation state in this isolated entity (see Tables S1 and S2†). Thus, this confirms the composition of anion 1. The result is rather surprising given the fact that previous reports revealed that tri-Ni substituted {PW_9_Ni_3_} moieties exist always in the form of aggregates or combine with other fragments to form derivatives,^23^ while isolated {PW_9_Ni_3_} anions yet remained unreported. A possible explanation for the formation of such a structure is the introduction of trivalent Ni^3+^ cations as further confirmed by other characterisation studies (see below), which stabilizes the highly negatively charged but unstable Keggin ion framework,^51,52^ indicating that the counter charge stabilization is crucially important to form the tri-Ni-POM structure compared with their all-Ni^2+^ counterparts.

**Fig. 2:**
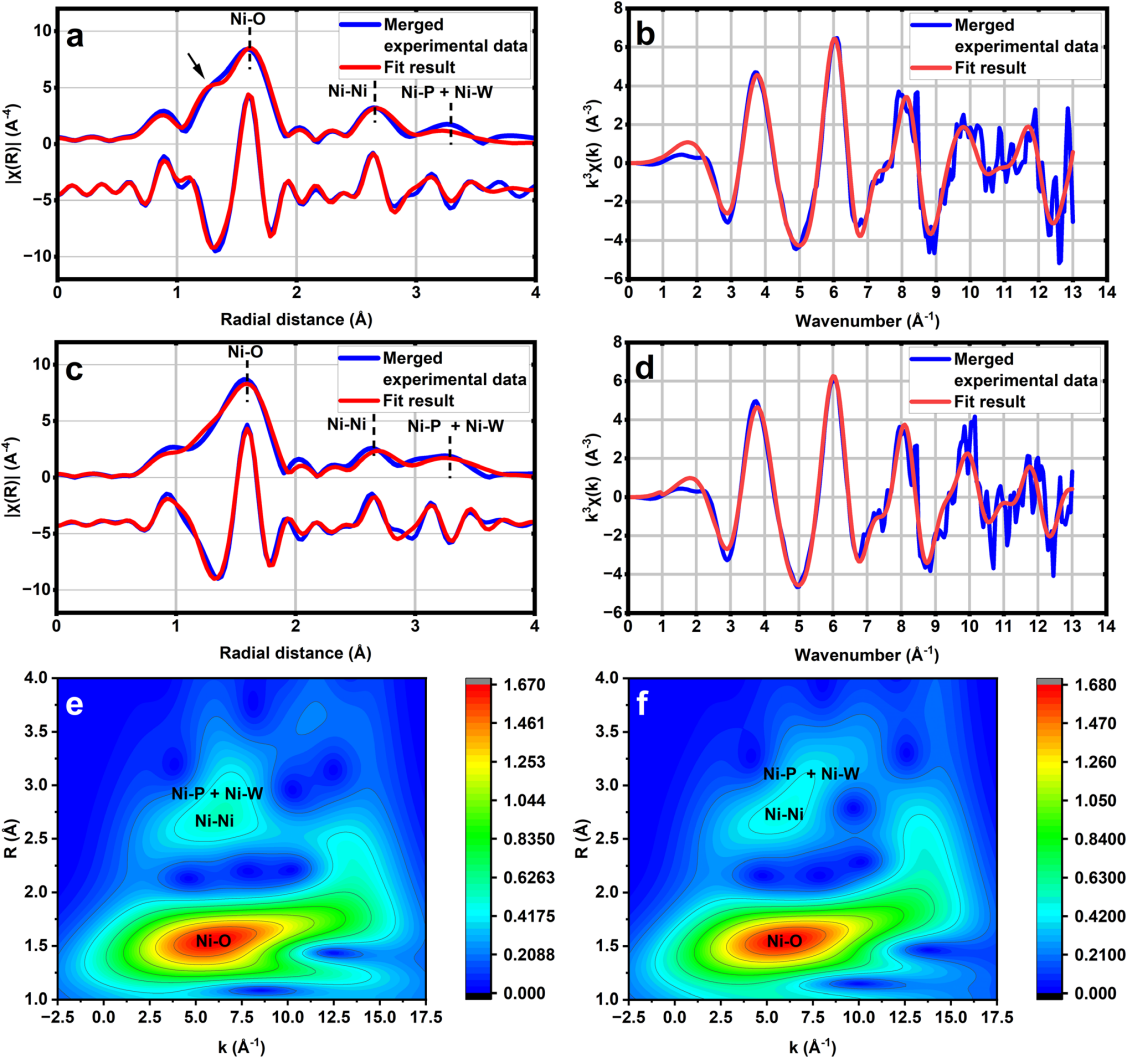
(a) Comparison of experimental (blue) and simulated (red) *k*^3^-weighted phase shift-corrected Fourier transformed EXAFS result of Cs_4_K-1 at the Ni K edge, including the magnitude (above) and real part (below), showing a distinctive shoulder peak in the Ni–O region (marked with an arrow). (b) Comparison of experimental (blue) and simulated (red) EXAFS chi functions of Cs_4_K-1 in *k* space at the Ni K edge. (c) Comparison of experimental (blue) and simulated (red) *k*^3^-weighted phase shift-corrected Fourier transformed EXAFS result of Cs_4_K-1 at the Ni K edge, including the magnitude (above) and real part (below). (d) Comparison of experimental (blue) and simulated (red) EXAFS chi functions of Cs_4_KH-2 in *k* space at the Ni K edge. (e) EXAFS WT 2D plots for Cs_4_K-1. (f) EXAFS WT 2D plots for Cs_4_KH-2.

Solid Cs_4_K-1 is expected to be highly oxidative but slowly transforms into a pale yellow or pale green solid Cs_4_KH[PW_9_Ni_3_(OH)_3_(OH_2_)_3_]·5H_2_O (Cs_4_KH-2) kinetically within a period of 2–4 months at 0 °C and about 1 month at room temperature (see Fig. S1†), accompanied by a continuous change of the characteristic P–O vibration peak in the IR spectrum. The reduction process of 1 seemed to be able to be accelerated when dissolved in water to form an aqueous solution (see below). In the case of Cs_4_K-1 a strong P–O stretching peak at around 1045 cm^−1^ with a small shoulder peak at 1082 cm^−1^ can be observed, along with characteristic peaks at 1045, 940, 883, 802 and 712 cm^−1^, respectively, which is in accordance with the substituted Keggin-type POM.^13,51,53^ As the solid reduces to form Cs_4_KH-2, the shoulder peak at 1045 cm^−1^ of 1 slowly vanishes while two new peaks at 1033 and 1057 cm^−1^ at lower wavenumbers slowly emerge, as shown in Fig. 1b. It's been confirmed that the P–O vibration peaks are coupled with the vibration of adjacent metal–oxygen bonds, and the weakening of ‘Ni–O’–P interaction due to the reduction from Ni^3+^ to Ni^2+^ may be responsible for the downshift and splitting of the peaks observed with lower symmetry.^54^ The position and shape of all the other peaks have a very good fit with that of the previously reported POMs with {PW_9_Ni_3_^II^} moieties,^55,56^ indicating that 2 also has a similar structure.

A noteworthy detail is that the position and splitting of P–O vibration peaks are therefore sensitive to the structural type of polyoxometalates, especially for the two most common types of PW_9_M_3_ moieties, namely A and B isomers (Fig. S2†). Generally speaking, the P–O vibration bands of B-PW_9_M_3_ Keggin moieties are not significantly split (smaller than 30 cm^−1^), while those of A-type isomers show a stronger band at higher wavenumber and a weaker band at lower wavenumber with a gap of greater than 50 cm^−1^, as revealed by previous literature of different PW_9_O_34_ derivatives.^13,51,57–59^ From this perspective, the IR spectra of both 1 and 2 fit well with the characters of B-type isomers, suggesting that both could adopt the same structure. ESI-MS analysis of samples of Cs_4_KH-2 (Fig. S3†) showed a series of mass spectroscopy peaks corresponding to [PW_9_Ni_3_^II^O_40_H_*x*_]^(15−*x*)−^ (*x* = 9, 10), indicating that 2 is an isolated tri-Ni-substituted Keggin-type POM similar to 1, and that the transformation from 1 to 2 is without significant structural change. However, compared with the mass spectrum of Cs_4_K-1, these main peaks are accompanied by various peaks corresponding to a series of {PW_9_Ni} or {PW_9_Ni_2_} anions, presumably formed by hydrolysis or fragmentation of anion 2 during its storage, implying the lower structural stability of 2 compared with 1.

The compositions of both Cs_4_K-1 and Cs_4_KH-2 were further confirmed by elemental analysis, which showed a slightly larger amount of metal elements in the compounds compared with the theoretical value, due to the loss of small amounts of crystallization water during the preservation of samples. Thermogravimetric analysis (Fig. S4 and S5†) of both compounds showed a major weight loss in the range of 100–200 °C corresponding to the loss of oxygen from the reduction of Ni^3+^ to Ni^2+^, and the loss of crystallisation water. A small weight loss was observed in both cases at 500 °C which may be attributed to the dehydration of the polyoxometalate anion. The result is in good agreement with a tri-nickel-substituted POM anion with 5 negative charges for 1 and 6 negative charges for 2.

Unfortunately, both Cs_4_K-1 and Cs_4_KH-2 consistently showed resistance towards crystallisation, and despite some weak diffraction peaks that can be observed in the powder XRD pattern, the solid products are generally of very low crystallinity, showing significant features of the amorphous phase (Fig. S6 and S7†). While Na^+^ and K^+^ salts of anion 1 without Cs^+^ seemed to be too soluble to precipitate, addition of other counter cations such as tetrabutylammonium (TBA) cations to the solution of Cs_4_K-1 does not give different products and does not help in getting better crystals, indicating that the additives are not involved in the crystallisation process. Thus, a combined approach of structural analysis and theoretical was used to identify the structure of the anions. The structures of a series of possible isomers of 1 were optimised, and the corresponding energies were obtained based on DFT calculations (see Fig. S2, Tables S3, S5–S11†). Among all isomers considered, the B-α-1 structure (Fig. 1c) has the lowest energy. The simulated IR spectra of corresponding isomers with different degrees of protonation, confirmed by computational results in two different combinations of basis sets, have a better fit with the experimental result when compared with the second most stable isomer, namely A-α-1 (Fig. S8–S11†). This is in line with the similarity of the IR spectra of 1 and 2 to other B-type isomers as discussed above. A similar computational result is obtained for 2 where B-α-2 is more energetically favoured compared with the speculated A-α-2 isomer (Tables S4, S12 and S13†). As the A-α isomer of [PW_9_O_34_]^9−^ was used as the reactant in the synthesis of Cs_4_K-1 and Cs_4_KH-2, this result would highlight a structural isomerisation of the {PW_9_O_34_} moiety from the A-α-type structure to its B-α-type isomer in the reaction. Interestingly, similar phenomena are also widely observed in other previously reported Ni-containing POMs,^53,54^ indicating that the relative structural stability of the B-α isomer compared to A-α structure in the presence of nickel ions is common. Notably, the Ni_3_ cluster in the structure of B isomers is very similar to the Ni_3_ fragments that can be found in the structure of Ni(OH)_2_ and NiOOH^43^ (Fig. 1d), hinting at a structural correlation between these molecular clusters and hydroxide compounds.

The structures of 1 and 2 are further studied by X-ray pair distribution function (XPDF) and extended X-ray absorption fine structure (EXAFS) analysis of Cs_4_K-1 and Cs_4_KH-2 (Fig. 2, S12 and S13†). The XPDF patterns of both compounds display various peaks, which may be attributed to different bonds based on the computational results, as shown in Fig. S12 and S13.† The strict fitting to the structural models would be impossible, as the very disordered crystallisation water and counter cations cannot be included in our structural model; thus, we compared the experimental spectrum with the simulated XPDF result based on the corresponding A and B isomers, respectively. In both cases, again, the simulated patterns of B isomers are closer to the experimental pattern in the range corresponding to Ni–Ni, Ni–W and W–W distances. The differences between the simulated spectrum and experimental spectrum in the range of 2.8–3.5 Å are likely to be attributed to ionic bonds between oxygen atoms and counter cations Cs^+^ and K^+^, with an estimated average K–O distance of approximately 3.0 Å and Cs–O distance of 3.3 Å and 3.7 Å, in good agreement with the previously reported results of polyoxometalates.^60^

To confirm the structural analysis result mentioned above, we further performed synchrotron X-ray absorption spectroscopy analysis on samples of Cs_4_K-1 and Cs_4_KH-2 to verify the short-range order in their structure. The optimised structure of B-α-1 has been fed into the IFEFFEIT program in the Artemis software package.^61^ Single scattering paths generated from this program are then shown to fit the extended X-ray absorption fine structures (EXAFS) part of the spectrum (Fig. 2, Tables S14 and S15†) with low R-factors (2.63% for 1 and 2.02% for 2), showing a good fit to both raw data (Fig. 2b and d) as well as the Fourier-transformed results (Fig. 2a and c). The Ni–O coordination number is evaluated to be 5.6 (6) with an average distance of 2.00(1)Å, aligning with the expectation of an octahedrally coordinated Ni centre. Careful examination of the Ni–O peak shows a significantly large shoulder at a shorter distance. According to the literature, Ni^3+^–O has a bonding distance of 1.94 Å and Ni^2+^–O has 2.07 Å,^62^ which matches the co-existence of Ni^3+^ and Ni^2+^ for the B-α-1 structure. The coordination number of Ni–Ni, Ni–P and Ni–W was fixed at 2, 1, and 2, respectively, during data analysis to obtain the corresponding radial distances, and the result has again, a very good fit with the corresponding value obtained from the B-α-1 structure which is characteristic for the B-type isomer of 1. A similar case is observed in the case of anion 2, for which the Ni–O coordination number is determined to be 5.7(8) at a longer average bonding distance of 2.03(1) Å, also in line with an octahedrally coordinated Ni. However, the shoulder peak region is much attenuated. Similar intermetallic radial distances were observed in the case which is also close to the computation value for the B-α-2 structure. Wavelet transformation (WT) analysis of the EXAFS patterns of both compounds clearly shows the existence of the second-shell coordination environment corresponding to Ni–Ni peaks. The Ni–P + Ni–W peaks with larger radial distances than Ni–Ni peaks can also be observed in both cases, which is in line with the structural feature of the B-type isomer. In general, the EXAFS WT plots of the two compounds are closely related to each other, suggesting the isostructural nature of the two anions as expected. Evidently, the B-type structure for both 1 and 2 can be affirmed by the strong correlation between their structure and EXAFS refinement results. Their clear differentiable and characteristic feature is the split of the Ni–O peak to give a shorter Ni–O distance (shoulder at shorter distance) indicative of the presence of Ni^3+^ in 1 from 2.

### Properties of the Ni^3+^ centres in tri-Ni POM, 1

As the initial studies have revealed the existence of Ni^3+^ centres in 1, more characterisation experiments were conducted to understand the electronic properties as well as the chemical behaviours of the speculated catalytically active high-valent Ni centres embedded in HPM frameworks. As shown in Fig. S14a,† the X-ray photoelectron spectroscopy (XPS) of Cs_4_K-1 clearly showed two components of nickel with the 2p_3/2_ peaks at 857.7 eV and 855.8 eV, respectively, assigned to Ni^3+^^63^ and Ni^2+^. It should be noted that the Ni^3+^/Ni^2+^ ratio calculated from the relative peak area is much smaller than the theoretical value of 2 : 1. This is due to the reduction of surface Ni^3+^ by atmospheric water or other reductive components during handling. This surface ratio is different from those of Ni^3+^/Ni^2+^ in the bulk phase obtained by other characterization methods such as magnetic measurements (see below) which is closer to the theoretical value. Nevertheless, the XPS result has undoubtedly confirmed the co-existence of Ni^3+^ and Ni^2+^. Interestingly, the binding energy values observed for both Ni^2+^ and Ni^3+^ are apparently close to the value measured for Ni(OH)_2_ and NiOOH, indicating that the chemical environment of these Ni centres could be comparable to their corresponding hydroxides, which are also known to be catalytically active towards water oxidation reactions^18^ and may indicate that these multi-Ni-substituted POMs could have similar WOC activity. This is understandable given that the chemical environment in polyoxometalate frameworks is similar to that of oxide systems,^64^ and that the structure of the Ni_3_ cluster proposed for 1 and 2 is similar to that of hydroxides.

In the corresponding spectrum of the reduced sample 2 (Fig. S14b†), the relative area of the 2p_3/2_ peak of the Ni^2+^ peak at 855.8 eV was significantly strengthened, with a very small peak at 857.7 eV assigned to very small amounts of unreduced Ni^3+^.

The magnetic properties of Cs_4_K-1 and Cs_4_KH-2 were also investigated by magnetic measurements (Fig. S15†), which revealed an approximate magnetic moment that can be estimated from the result as *ca.* 4.7–4.8 B.M based on the magnetization of Cs_4_K-1 in the range of 0–70 000 Oe, corresponding to two low-spin Ni^3+^ and one Ni^2+^ in compound Cs_4_K-1, which gives the theoretical spin-only value as 4.90 B.M. Similarly, an experimental value of 6.20–6.40 B.M. is observed for compound Cs_4_KH-2, while the theoretical spin-only value expected for a *S* = 3 Ni_3_^II^ cluster is 6.93 B.M. This good agreement between the postulation and the experimental result confirms the existence of {Ni_2_^III^Ni^II^} and {Ni_3_^II^} clusters in 1 and 2, respectively. This result is also in line with the ESI-MS result, which further proves the existence of high-valent Ni^3+^ in 1.

**Fig. 3:**
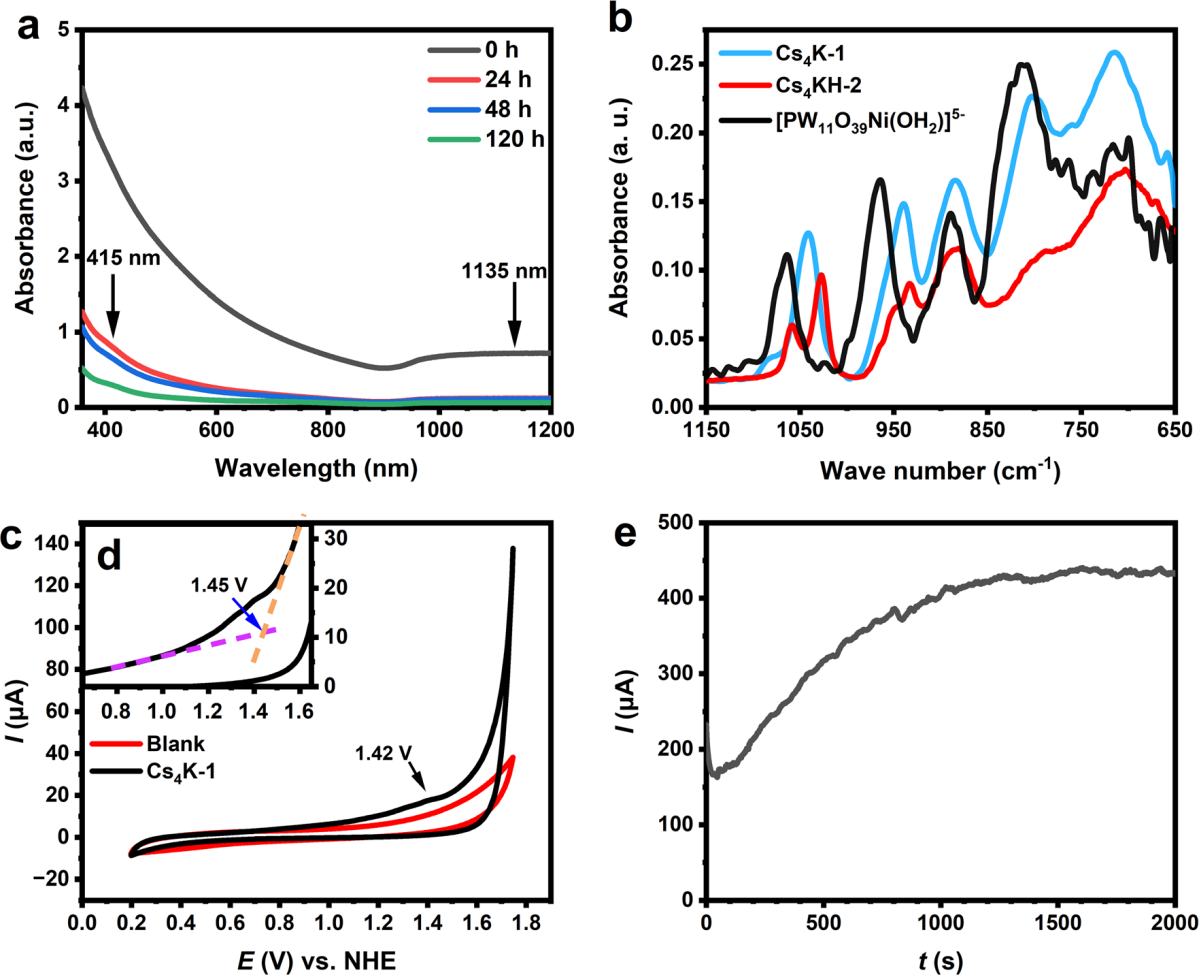
(a) Time-dependent UV-Vis spectra of Cs_4_K-1 in aqueous solution in the range of 350–1200 nm. (b) Comparison of the IR spectrum of salts of [PW_11_NiO_39_(OH_2_)]^5−^, Cs_4_K-1 and Cs_4_KH-2. (c) Cyclic voltammogram (CV) of Cs_4_K-1 in aqueous solution. The blank solutions are shown for comparison. (d) Illustration of the calculation of the onset potential of Cs_4_K-1. (e) Chronoamperometric curve of Cs_4_K-1 in 0.5 M Na_2_SO_4_ solution at 1.85 V *vs.* NHE.

The optical absorption properties of 1 in aqueous solution are studied by UV-Vis-NIR measurements. A freshly prepared solution of Cs_4_K-1 shows strong absorption in the range of 350–1200 nm with a broad absorption peak in the NIR region (*λ* > 950 nm) (see Fig. 3a, S16 and S17†). The experimental spectrum is close to a combination of simulated UV-Vis spectra by the time-dependent density functional theory (TD-DFT) method based on the structure B-α-1 and its protonated form (Fig. S16b and c†). It can be found that the experimental spectrum is similar to a combination of two computed spectra, and a broader absorption in the range of 600–800 nm is characteristic of the protonated form. An NIR peak at 1135 nm is observed in the simulated UV-Vis spectrum of 1 which corresponds to the experimentally observed NIR peak, and the peak does not occur in the protonated form (Fig. 3b). Detailed electron–hole analysis^45^ (Fig. S16d and e†) showed that d–d transitions of Ni^2+^/Ni^3+^ centres and ligand-to-metal charge transfer (LMCT) transition all contributed to the absorption in the visible region, while the peak at 1135 nm in the simulated spectrum of 1 corresponded to a coupled LMCT process from the hydroxyl ligand to the neighbouring Ni^3+^ centre and the d_*z*^2^_-to-d_*x*^2^−*y*^2^_ transfer process of the corresponding Ni^3+^. As a result, the occurrence of the 1050 nm peak is a clear indication of the existence of Ni^3+^ coordinated with hydroxyl ligands.

Cyclic voltammograms (CVs) of Cs_4_K-1 in a neutral aqueous environment show the irreversible oxidation peak at +1.42 V *vs.* NHE, which can be attributed to the oxidation of Ni^2+^ to Ni^3+^ (Fig. 3c and S18†). No corresponding reduction peak is observed in the CV. The corresponding peak was observed in a clearer manner in the differential pulse voltammogram (DPV) of Cs_4_K-1, showing the corresponding peak at 1.39 V *vs.* NHE (Fig. S19a†). The difference in the peak potential observed in CV and DPV is due to the fact that non-faradaic current was minimised in the measurement of DPV, while in CV both faradaic and non-faradic currents were included.^65^ A sharp increase in current density is actually observed beyond an onset potential of 1.46 V *vs.* NHE (Fig. 3d) with bubbles occurring on the electrode surface, indicating the voltage initiation of the electrochemical water oxidation process. The current is significantly larger than that of pure electrolyte solution, confirming the enhanced water oxidation activity in the presence of 1, as expected before. It is noteworthy that a weak oxidation peak is observed at 1.52 V *vs.* NHE in the DPV of Cs_4_K-1, which is just slightly higher than the onset potential value and might be the actual species obtained by further oxidation of anion 1 that is responsible for the water oxidation. Based on the structural similarity of 1 and hydroxide systems, we postulate that this species could be {Ni^III^–O˙}oxo-like species, similar to NiOOH.^66^ This is in line with the generally proposed catalytic mechanism for WOCs, and similar intermediates have also been proposed or studied in many similar POM-based WOCs.^9,67–69^

No redox peaks related to the reduction of W^6+^ centres were also found in the CV of Cs_4_K-1 in the range of −1.0 to 0.2 V *vs.* NHE. However, we have indeed found two pairs of redox peaks that are close to the reported W^6+^/W^5+^ redox peaks for substituted Keggin-type polytungstates in the DPV results.^23^ Thus, these peaks are assigned to the redox reaction of W centres (see Fig. S20†). The absence of these peaks in CV is probably due to their low peak current, which is also observed in the previously reported results for other Ni-containing polyoxometalates containing {PW_9_Ni_3_} moieties.^23^

### Stability of tri-Ni POMs, 1 and 2 in an aqueous environment

In spite of the observed high WOC activity of 1 in an aqueous environment, the stability of the anion is yet to be monitored in order to determine the “real” catalytic efficiency of the POM catalysts.

We first examined the stability of 1 towards spontaneous reduction in an aqueous environment without the application of potential. As shown in Fig. 3a, the characteristic broad absorption of 1 gradually decreases in a 120-hour period of measurement, accompanied by the fading of the solution from dark brown to pale yellow. The absorption spectrum of the solution after 120 hours showed characteristic d–d transition peaks of Ni^2+^ at 415 and 680 nm, respectively, similar to the simulated UV-Vis spectrum of 2 (Fig. S21†) and other known Ni^2+^ complexes including [PW_11_NiO_39_(OH_2_)]^5−^. No precipitate was observed in the solution of Cs_4_K-1 when it was kept at low temperatures or in the first few days, indicating its rather good hydrolytic stability of the anion without the use of potential. Note that the solid product collected by either precipitating the anion or evaporating from the solution showed no change in Cs_4_K-1, while attempts to re-obtain solid Cs_4_KH-2 from either its solution or reduced solution of Cs_4_K-1 only led to the formation of large yellowish-green crystals containing [PW_11_NiO_39_(OH_2_)]^5−^ accompanied by an unidentified precipitate. The identity of [PW_11_NiO_39_(OH_2_)]^5−^ is confirmed by its characteristic P–O stretching peak at 1064 cm^−1^ and W–O stretching peaks at 964, 889, 802, and 714 cm^−1^, in good agreement with previous literature.^70,71^

The observed phenomena are in line with the ESI-MS result, where no hydrolytic products were observed in the spectrum of Cs_4_K-1, but various fragmentary anions were observed for Cs_4_KH-2. This suggests the fact that 1 displays a more superior hydrolytic stability while 2 is significantly hydrolytically unstable. The formation of [PW_11_NiO_39_(OH_2_)]^5−^ as the POM product of the hydrolysis, suggests that hydrolytic decomposition on both tungsten and nickel sites, could result in a complete structural reorganisation of the original POM framework. Similar hydrolytic reactions on multiple sites have also been found in previously reported substituted POMs.^72^ The hydrolytic instability of 2 highlights the potential disturbance by WOC-active transition metal oxide or hydroxide species during activity evaluation of these substituted POM-based WOCs as reported in those cobalt-based POM WOCs.^16^

It is believed that the difference in hydrolytic stability of 1 and 2 could be attributed to the overall anionic framework stability in water. The stronger covalent (ligand field) and electrostatic interactions (ion-pair like) between polytungstate moieties and Ni^3+^ strengthen not only the bonding between Ni^3+^ and ligands but also between the whole tungstate units in this polytungstate ligand, preventing the POM framework from disintegration under aqueous conditions. In contrast, the weaker interaction between Ni^2+^ and polytungstate ligands may lead to lower hydrolytic and structural stability. A similar case is known for the cobalt-based POM WOC [Co^III^Co^II^W_11_O_39_]^7−^ with the oxidation state of the central Co centre being +3. The POM itself was found to be hydrolytically stable while its all-Co^2+^-substituted counterpart was significantly unstable. Despite this, the complex was reported to have good stability under catalytic conditions.^24^ Given the fact that better hydrolytic stability of 1 compared to 2, would the Ni^3+^-containing anion 1 show better stability in the water oxidation catalytic process?

The stability test of Cs_4_K-1 under electrochemical conditions in neutral aqueous solution by the chronoamperometric (CA) method at 1.85 V *vs.* NHE, shows a continuously increasing current after 25 s and reaches a rather stable current after 1500 s (Fig. 3e). We noted that such a pattern is similar to the CA curve of some hydrolytically unstable transition metal-substituted polyoxometalates,^73^ which formed oxides on the surface of electrodes leading to an increasing chronoamperometric current curve. The CV changes significantly in the range of 1.2–2 V *vs.* NHE after the test (Fig. S22†) while a layer of black substances, tentatively assigned to NiO_*x*_ containing precipitate, was found deposited on the surface of the electrode. By evaporating the solution after the electrochemical test, salts containing [PW_11_NiO_39_(OH_2_)]^5−^ were identified, indicative of a hydrolytic decomposition process of anions 1/2 with the global structural change of the POM species and the release of Ni^2+^. This result is apparently in contrast to the observed hydrolytic stability of 1 as confirmed before, and is more likely to be attributed to the hydrolysis of 2*via* its formation as an intermediate in the process. The heterogeneous hydrolytic product is postulated to be the main contributor to the large water oxidation current observed in the CA curve after 25 s, the formation rate of which is apparently much faster than the spontaneous reduction of 1 in solution without applying voltage. This indicates a significant acceleration under such electrochemical conditions, and cannot be explained by the formation of 2 from the spontaneous reduction process of 1 without the use of potential. Two conclusions can therefore be drawn from the above observations: (i) the reduction and/or hydrolysis of the POM WOC species in the solution, either 1 or 2, are significantly accelerated under the applied voltage, likely due to the involvement of 2 in the water oxidation catalytic cycle: this explains why the synthesis of Cs_4_KH-2 and related tri-Ni-substituted Keggin-type polyoxometalates was previously unsuccessful due to the instability of 2; (ii) the result also suggests that the intermediates/products formed in catalytic cycles may critically affect the evaluation of activity (initial activity must be used) and stability of POM WOCs.

### Stabilisation of tri-Ni POM, 1 and 2 redox pair by the MIL-101(Cr) framework

The relative hydrolytic instability of 1 and 2 and the failure of their recycling present a fatal challenge to the potential application of these multi-Ni-based POM clusters to be used as WOCs. Thus, a synthetic strategy must be developed to stabilise these POM clusters for application purposes. Appropriate stabilisation of these POM species would enable them to be used as viable catalysts for practical water oxidation processes.

**Fig. 4:**
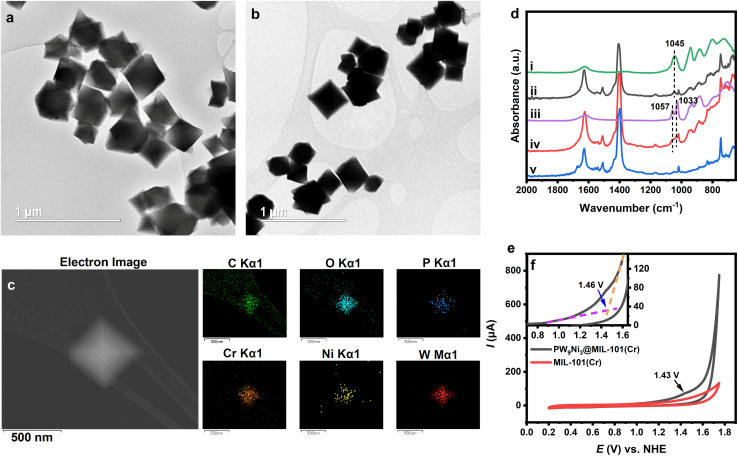
(a) Bright-field-conventional transmission electron microscopy (BF-CTEM) images of the PW_9_Ni_3_/MIL-101(Cr) composite. (b) (BF-CTEM) images of MIL-101(Cr) particles. (c) Dark-field-scanning transmission electron microscopy (DF-STEM) image of a PW_9_Ni_3_/MIL-101(Cr) crystallite with the corresponding energy-dispersive X-ray spectroscopy (EDS) mapping result of the composite carbon (C), oxygen (O), phosphorus (P), chromium (Cr), nickel (Ni) and tungsten (W). (d) Comparison of IR spectra of Cs_4_K-1 (i), the PW_9_Ni_3_/MIL-101(Cr) composite after 20 min of sonication (ii), Cs_4_KH-2 (iii), the composite after 24 hours of stirring (iv) and MIL-101(Cr) (v). (e) Cyclic voltammogram (CV) of the PW_9_Ni_3_/MIL-101(Cr) composite and MIL-101(Cr) in 0.5 M Na_2_SO_4_ solution. (f) Illustration of the calculation of the onset potential of the PW_9_Ni_3_/MIL-101(Cr) composite.

Some methods have been previously identified empirically to be effective in preventing POM species from hydrolysis, such as precipitation with counter cations or incorporation into porous frameworks. It is well-known that polyoxometalates can be preserved against hydrolytic decomposition by incorporation into MOFs, and such composites have been widely tested as heterogeneous WOCs with promising stability and activity.^74–76^ We attempted to use MIL-101(Cr)s which are positively charged [Cr_3_O(OH_2_)_3_(bdc)_3_]_*n*_^*n*+^ framework cations (bdc = terephthalate anion ligands) counter-balanced by small anions, hence they could bind with the negatively charged polyoxometalate anion.^74^ It is also well-known for its large pore size sufficient to entrap polyoxometalate anions, acceptable electric conductivity and redox inertness under high potential.^73^ As a result, MIL-101(Cr) would be an ideal choice for the heterogenization and stabilization of 1 and 2. Thus, the composite based on MIL-101(Cr) was synthesised by impregnating MIL-101(Cr) in the aqueous solution of 1. The composite shows characteristic IR features of 1 after 20 min of sonication (Fig. 4d and S23†), indicating the successful incorporation of such an anion with Ni^3+^ and Ni^2+^ into the pore of MIL-101(Cr). Upon extending the stirring time period to 24 h at room temperature, the Ni species has apparently been reduced to 2 that mainly contains Ni^2+^ inside the entrapped environment, as shown by its characteristic peaks in the IR spectrum as Cs_4_KH-2 (Fig. 4d and S24†).^8^ The resulting composite precipitation can be easily separated from the solution by centrifugation. The composite showed a powder XRD pattern very similar to the original MIL-101(Cr) framework but with a change in the relative intensity of the diffraction peaks (Fig. S25†). This indicates the fact that although the crystallinity is mainly preserved, the stereospecific entrapment of polyoxometalate inside the pore altered the diffraction intensities.^74,77^ The Ni k-edge EXAFS pattern of the composite showed a similar pattern to the EXAFS pattern of Cs_4_KH-2 (Fig. S26, Table S16†), which can again be fitted by the structure of B-α-2 in a satisfactory manner. The WT analysis of the EXAFS result also showed a second shell Ni–Ni peak and Ni–P + Ni–W peak similar to the case of Cs_4_KH-2. The oxidation state of nickel centres in the final composite was further confirmed by XPS analysis of the composite which showed that all nickel atoms are in the +2 oxidation state (Fig. S27†). Different from a homogeneous solution of Cs_4_KH-2 in which anion 2 is rapidly hydrolysed to form [PW_11_NiO_39_(OH_2_)]^5−^, the stability of 2 in the composite after being stirred in an aqueous environment for 24 h was kinetically ensured, confirming the effective protection of the hydrolysis-vulnerable anion 2 against decomposition by the MIL-101(Cr) framework (Fig. 4d).

Transmission electron microscopy (TEM) shows the near-perfect octahedral morphology of MIL-101(Cr) which is also well preserved in the composite (Fig. 4a and b). Energy-dispersive X-ray spectroscopy (EDS) elemental mapping of the composite (Fig. 4c, S28 and S29†) shows evenly distributed elements: P Kα-signal at 2.013 keV, W M-signal at 1.774 keV and L_α_-signal at 8.396 keV, Ni L_α_-signal at 0.851 keV and Kα-signal at 7.471 keV, respectively. The EDS also confirms the composite matching with [Cr_3_O(OH_2_)_3_(bdc)_3_]_6_[PW_9_Ni_3_O_34_(OH)_3_(OH_2_)_3_)] which contains nickel in the +2 oxidation state. The 6 : 1 ratio of MIL-101(Cr) to PW_9_Ni_3_ species is also consistent with anticipated electrostatic binding and stabilization between the cationic framework and anionic polyoxometalate in this composite.^77^ Meanwhile, the Brunauer–Emmett–Teller (BET) curves of MIL-101(Cr) and the composite are shown in Fig. S30† with the surface area and pore volume data listed in Table S17.† The significantly reduced surface area and pore volume compared with pure MIL-101(Cr) also confirm the existence of polyoxometalate anions in the MIL-101 framework. The space around the entrapped polyoxometalate 2 is rather limited as reflected by the pore distribution analysis (Fig. S31†) with a significant decrease in pore diameter to approximately 18 Å. This is thought to offer severe spatial restrictions to its hydrolytic decomposition but further studies on hydrolytic mechanism and corresponding structural rearrangement within the pore are required.

**Scheme 1:**
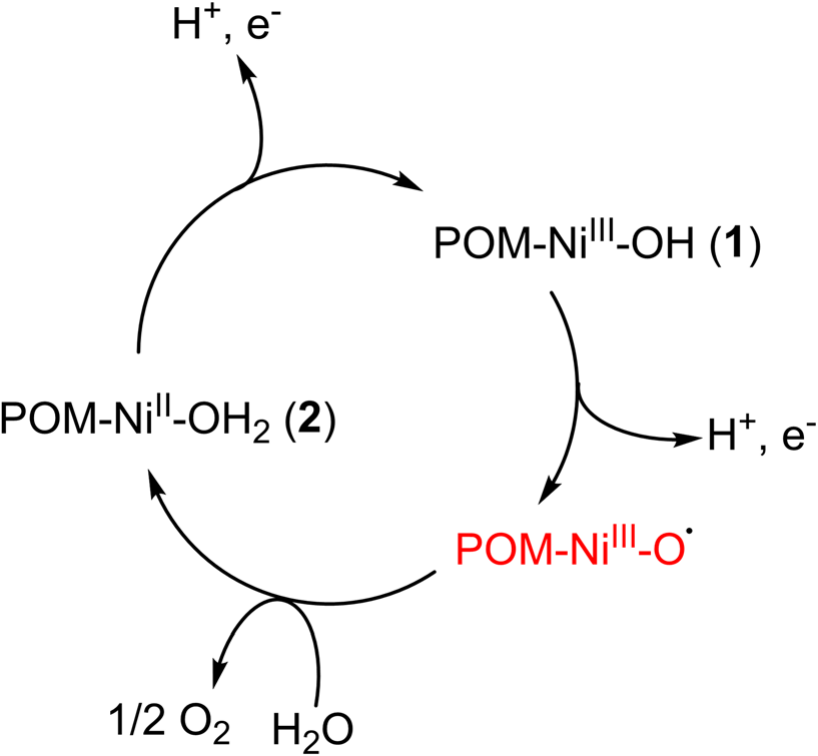
Proposed water oxidation catalytic cycle of 1/2 entrapped redox pair under electrochemical conditions.

To confirm the stabilization of the 1 and 2 redox pair inside the MIL-101(Cr) framework, the electrochemical properties of the composite were investigated using composite-loaded carbon paper as a working electrode in the neutral aqueous environment. The CV of the composite showed a very similar initial pattern compared with free 1 in the aqueous solution (see Fig. 4e), indicating the same conversion process between 1 and 2. The irreversible oxidation peak of Ni^2+^ occurred at the potential of approximately 1.43 V *vs.* NHE along with a significant high water oxidation current starting from 1.46 V. This indicates that the oxidation of 2 to 1 is indeed part of the catalytic cycle in water oxidation. The onset overpotential for the OER is thus calculated to be 0.64 V. This value is comparable to that of various POM-based WOCs and the composite has shown better performances when compared to some POM WOCs such as [CoW_12_O_40_]^6−^, thus illustrating the advantages of using PW_9_Ni_3_-based WOCs (see Table S18†).^15,78–80^ The similarity of CV features between Cs_4_K-1 and the composite is also observed in DPV with a broader peak at 1.39 V *vs.* NHE. The 1.52 V peak observed in the DPV of Cs_4_K-1, which may correspond to the formation of {Ni^III^–O˙} species, seemed to have merged into the broadened peak at 1.39 V in the case of the composite (Fig. S19b†). The similarity of CVs of Cs_4_K-1 (mainly containing anion 1) and the composite (mainly containing anion 2), along with high water oxidation current after the oxidation peak of Ni centres confirm the involvement of both 1 and 2 in the catalytic cycle. The exciting stability of the entrapped catalyst by our synthetic strategy is therefore clearly demonstrated. Based on these results, a catalytic mechanism is proposed as shown in Scheme 1.

In comparison, carbon paper electrodes loaded with MIL-101(Cr) did not show any water oxidation activity. The cyclic voltammetry test of the composite in a larger range of −0.3 to 2.0 V (Fig. S32a†) was also similar to the CV of aqueous solution of 1 in the same range, with a significant ORR current occurring at lower potential (Fig. S32b†). The Cr^2+^/Cr^3+^ redox peaks in the MIL-101(Cr) framework were observed at around 0.3 V which is in good agreement with the literature value.^81^

The electrochemical water oxidation catalytic activity of the composite loaded on carbon paper electrodes was evaluated by the linear scan voltammetry (LSV) technique, and the result is shown in Fig. S33.† The composite showed significantly increasing water oxidation current after applying the onset potential, while MIL-101(Cr) did not show any water oxidation activity. This implies that the WOC activity of the composite originates from the nickel-substituted polyoxometalates rather than from the framework itself. Interestingly, the onset potential of the 1 and 2 redox pair is apparently higher compared with the previously reported cobalt-based POM WOCs,^4^ and this is also in line with the relationship of onset potentials between Co- and Ni-based hydroxide WOCs,^82^ emphasising the similarity between molecular POM WOCs and bulk heterogeneous hydroxide systems, as shown by the structural features and the XPS result. The Tafel slope estimated based on the LSV curve is 376 mV dec^−1^, which is much higher than the value obtained in the aqueous solution measurement of 1. This high Tafel slope can be explained by the high internal resistance of the porous MIL-101 framework due to its poor electrical conductivity, and a similar case of high Tafel slope has been reported for the SiW_12_/ZIF-8 polyoxometalate-MOF composite when being used as a WOC.^72^ This is further confirmed from the EIS Nyquist plot of MIL-101(Cr) loaded on carbon paper electrodes (Fig. S34†). Through the fitting of the equivalent circuit, a charge transfer resistance value of 1937.3 Ω could be obtained, indicating a significant barrier in electron transfer which is similar to that of ZIF-8-based materials.^83,84^ Despite this, the charge transfer resistance is significantly reduced to 584.6 Ω when PW_9_Ni_3_ clusters are entrapped in the MIL-101(Cr) framework to form the composites, thus confirming the enhanced electron transfer of the composite in the presence of the 1/2 redox pair.

The stability of the composite under electrochemical conditions was again monitored by CA measurement. As expected for the effective stabilisation of 2 towards hydrolysis, a steady *I*–*t* curve was obtained in the measurement period of 1 hour, with only slight fluctuation due to the formation of bubbles on the electrode surface, indicating the absence of NiO_*x*_ species.^16^ A slight drop in the initial current density was hereby observed, presumably due to the gradual leakage of those surface-attached polyoxometalate from MIL-101(Cr) before reaching the steady value (Fig. S35†). The current density after 1 hour of testing still reaches 90% of the initial value (1.3 mA cm^−2^). In contrast, the *I*–*t* curve of the raw MIL-101(Cr) framework showed a low and slightly increasing current during the measurement, which suggests a decomposition of the framework itself. Based on the result, two conclusions can be made: (i) the active centre of the composite as a WOC is the species incorporated, rather than MIL-101(Cr) itself; (ii) the formation of the composite does not only enhance the stability of the POM anion but also the MIL-101(Cr) framework itself, as confirmed by previous literature already.^74^

**Fig. 5:**
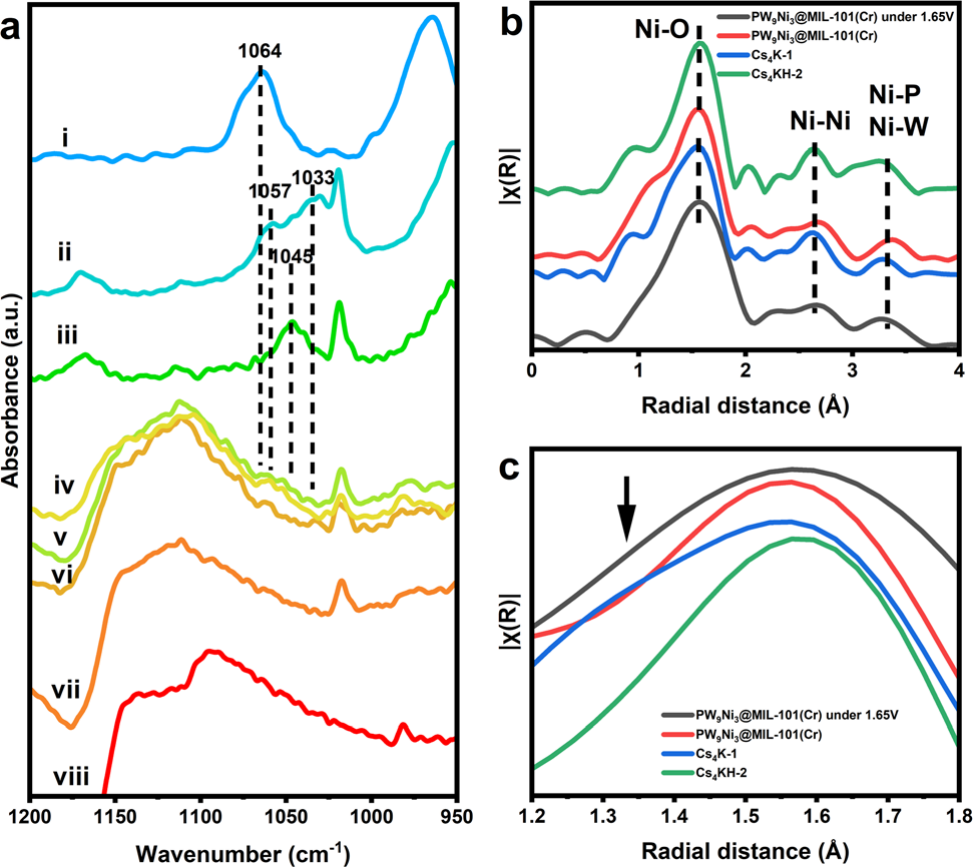
(a) Comparison of IR spectra of (i) salts of [PW_11_NiO_39_(OH_2_)]^5−^, (ii) PW_9_Ni_3_/MIL-101(Cr) composite (containing 2), (iii) PW_9_Ni_3_/MIL-101(Cr) composite after 20 min of sonication (containing 1), (iv)–(vi) measurements of different sites on the surface of the PW_9_Ni_3_/MIL-101(Cr) composite on the carbon paper electrode after the water oxidation test, (vii) MIL-101(Cr) on the carbon paper electrode after the water oxidation test, (viii) carbon paper electrode after the water oxidation test. (b) Comparison of EXAFS pattern of Cs_4_K-1, Cs_4_KH-2, and the PW_9_Ni_3_@MIL-101(Cr) composite and the *in situ* measurements of PW_9_Ni_3_@MIL-101(Cr) with an applied potential of 1.85 V *vs.* NHE. (c) Comparison of the Ni–O peak in the *k*^3^-weighted Fourier-transformed EXAFS pattern of Cs_4_K-1, Cs_4_KH-2, PW_9_Ni_3_@MIL-101(Cr) composite and the PW_9_Ni_3_@MIL-101(Cr) with an applied potential of 1.85 V *vs.* NHE. The position of the peak broadening/shoulder peak is marked with the arrow.

The IR spectrum (Fig. S36a†) after the reaction consistently showed the characteristic peaks of MIL-101(Cr), indicating the successful preservation of the framework structure. Noticeably, there is a broad vibrational peak at around 1100 cm^−1^, which was also observed in raw carbon papers after the same electrochemical treatment. Presumably, such a peak originated from the oxidation of carbon paper under water oxidation voltage (Fig. 5a and S36†). Identifiable P–O vibration peaks of PW_9_Ni_3_ species can still be clearly observed at 1030–1060 cm^−1^, as confirmed by multiple measurements on different sites on the electrode surface. The peaks generally match the signals of 1 and 2 but are distinctive and different from those P–O stretching peaks of the hydrolytic product, [PW_11_NiO_39_(OH_2_)]^5−^. Thus, it confirms the structural integrity of PW_9_Ni_3_ within the composite without any characteristic peaks of Ni(OH)_2_ and NiOOH at 3640 cm^−1^ (Fig. S36b†).^16^ The absence of the postulated hydroxide species further confirmed that the POM anion incorporated is the genuine guest species incorporated in the MIL-101(Cr) framework rather than the NiO_*x*_ species, and hence the real active centre of the water oxidation catalytic process. It should be noteworthy that the stability observed here does not conflict with the instability of anion 1 towards spontaneous reduction as the main species in the composite is expected to be anion 2.

Interestingly, the IR spectra also show peaks at around 1045 cm^−1^, more akin to 1. This suggests that entrapped 2 can be re-oxidised to 1 during the water oxidation cycle, indicating the co-existence of 1 and 2 under electrochemical conditions at a kind of dynamic equilibrium. It is envisaged that the use of high potential could cycle them in accordance with the proposed catalytic cycle in Scheme 1. Fig. 5b and c show the *in situ* EXAFS measurements of the composite with and without applied potential of 1.85 V *vs.* NHE. As previously stated, Ni^3+^ in 1 gives a clear differentiable and characteristic shoulder at a shorter radial distance in the Ni–O region in the FT-EXAFS (refer to Fig. 2).^57^ At the applied potential of 1.85 V, the composite showed a similar EXAFS pattern to the composite without applied potential, but showed a broadened Ni–O peak indicative of partial formation of a shoulder peak, suggesting the structural stability of 1 under water oxidation conditions and the electrochemical driving potential for oxidation in the aqueous phase.

As a result, the observed stabilization of entrapped tri-Ni-substituted Keggin-type polyoxometalates may be attributed to the strong host–guest interactions within the MIL-101(Cr) framework, characterized by envisaged hydrogen bonding, electrostatic interactions and specific spatial entrapment, which strengthen the structural integrity of both the MOF and POM, preventing them from being disintegrated and reorganised during catalysis under extreme conditions.^76,85^ In this respect, it's intrinsically similar to the relative stability of 1 compared to 2, which is attributed to limited degrees of electrostatic interactions in their ion-pairing. The successful stabilisation of intrinsic unstable 2 in MIL-101(Cr) and its facilitated recycling to and from 1 in the catalytic cycle at the applied potential has led to significantly improved catalysis. This is further proven by the CV curve of the composite after further extending the OER test to 10 000 seconds (Fig. S37†) which showed no significant change in the shape, revealing that the composite was retained without decomposition or formation of new species even after elongated measurement period. Despite this, it should be noted that a current decrease of 16% was observed at 1.85 V *vs.* NHE (OER measurement potential) in the CV of the composites, suggesting further leaching of POMs from the MIL-101(Cr) framework. Similar phenomena have been observed in other POM-MOF composites due to the continuous leakage of POM anions,^74,76^ indicating that leaching of the POMs from the framework under electrochemical conditions still remains a main issue for the MOF-entrapped POM materials, including the PW_9_Ni_3_/MIL-101(Cr) composite, as long-lived WOCs. A potential solution that has been explored is the one-pot “bottle-around-a-ship” synthesis method,^76^ and we plan to explore the strategies to apply the one-pot method to more hydrolytically unstable POM clusters *via* redox reactions in further studies.

## Conclusions

By studying the structural, chemical and stability of a pair of isostructural multi-nickel-containing POMs with variable oxidation states of Ni ions, a greater depth understanding of their potential application as WOCs can be obtained. Our research here also reveals the redox properties of Ni^3+^/Ni^2+^ as co-operative ‘tri-Ni’ transition metal ions in the POM framework, which are the key in such highly active molecular WOCs. The construction of unprecedented isolated {PW_9_Ni_3_} by using the oxidation method associated with silver has also inspired us with the new charge tailored synthetic strategy towards stabilized POMs in MOFs as new WOCs. Through appropriate stabilisation and *in situ* redox reactions, one can expand the palette of substituted POM-based molecular WOCs with new, catalytically active structures unobtainable by direct one-pot reactions.

In short, we have highlighted the possibility that the incorporation of POM clusters into MOFs such as MIL-101(Cr) is a promising solution in solving the long stabilization issue in developing POMs for WOC or related catalysis under extreme conditions. This approach could be useful in the further development and exploration of more POM-based WOCs. We expect to expand our exploration in optimising the corresponding catalytic systems to obtain more stable and effective POM-based WOCs.

## Data availability

The additional data are provided in the ESI.† All the data that support the findings of this study are available from the corresponding author upon reasonable request. Source data are provided in this paper.

## Author contributions

X. L. performed the preparation, testing and characterization of catalysts with assistance from C. J. and J. S; P.-L. H. collected the HRTEM data; B. N., T.-S. W. and Y.-L. S. performed the XAS characterization; T. Y. and X. P. performed the XPDF and *in situ* XAS analysis; H. H., B. Y. and S. C. E. T. were responsible for research directions; S. C. E. T. and X. L. wrote the manuscript with help from all authors.

## Conflicts of interest

There are no conflicts to declare.

## Supplementary Material

SC-015-D4SC01087F-s001
