# Supplementary material for: Stabilization of Ni-containing Keggin-type polyoxometalates with variable oxidation states as novel catalysts for electrochemical water oxidation
Source: Chem Sci. 2024 May 13;15(24):9201–15. doi: 10.1039/d4sc01087f (PMC11186315; doi:10.1039/d4sc01087f)
Supplement: SC-015-D4SC01087F-s001 [file SC-015-D4SC01087F-s001.pdf]

Electronic Supplementary Material (ESI) for Chemical Science.
This journal is © The Royal Society of Chemistry 2024

Supporting Information

Stabilization of Ni-containing Keggin-type Polyoxometalates with Variable Oxidation States as Novel Catalysts for Electrochemical Water Oxidation

Xiang Li[a], Bryan Ng[a], Ping-Luen Ho[a], Chunbo Jia[b], Jining Shang[b], Tatchamapan Yoskamtorn[a], Xuelei Pan[a], Yiyang Li[a], Guangchao Li[a], Tai-Sing Wu[c], Yun-Liang Soo[c], Heyong He[b]*, Bin Yue[b]*, S.C.E Tsang[a]*

[a]*Department of Chemistry, University of Oxford, Oxford, OX1 3QR, United Kingdom*

[b]*Department of Chemistry, Fudan University, Shanghai, China*

[c]*National Synchrotron Radiation Research Center, 101 Hsin-Ann Road, Hsinchu 30076, Taiwan*

Experimental Section

Electrospray ionisation mass spectrometric measurement

The mass spectrometric measurements were performed on a Bruker MicrOTOF II mass spectrometer in the m/z range of 300-2500 with a capillary voltage of 4000 V and an electrospray ionisation (ESI) source. The mode was negative ion. The nebuliser pressure was 1.0 bar and the dry heater temperature was 180 ºC with a dry gas flow of 4.0 L·min^{-1}.

BET analysis

BET analysis was conducted by N_2 adsorption method measured at 77 K and the pressure up to 1 bar. The isotherm was recorded on a Micromeritics Tristar instrument. All sorption isotherms were obtained using ultrahigh purity gases (99.999%). Before the sorption analysis, a sample (0.05−0.1 g) was loaded into a sample cell and subjected to a vacuum of 10−5 Torr at 423 K for 12 h. N_2 adsorption data with an initial slope (0.01 to 0.1 P/P_0) permitted calculation of the apparent surface areas based on the Brunauer-Emmett-Teller (BET) equations. Pore size distribution (PSD) was calculated by DFT embedded in a Micromeritics software. A model used in the PSD analysis is based on a cylindrical pore structure with oxide surface analyzed by N_2 at 77 K.50mg of MIL-101(Cr) and 100mg of PW_9Ni_3/MIL-101(Cr) composite were used for the analysis respectively.

Extended X-ray Absorption Fine Structure (EXAFS) analysis

X-ray absorption spectroscopy (XAS) data of **Cs_4K-1** and **Cs_4KH-2** was measured at beamline TLS07A of Taiwan Light Source at National Synchrotron Radiation Research Centre (NSRRC). XAS data of PW_9Ni_3/MIL-101(Cr) composite was measured at beamline BL11B of Shanghai Synchrotron Radiation Facility (SSRF). Fluorescence mode was used for Ni K-edge measurements and achieved by using a silicon drift detector. To ascertain the reproducibility of the experimental data, at least 3 scan sets were collected and compared for each sample.

The EXAFS data analysis was performed using IFEFFIT with Horae packages (Athena and Artemis)[3]. The spectra were calibrated with Ni metal foil as a reference to avoid energy shifts of the samples. And the amplitude reducing parameter was obtained from EXAFS data analysis of the Ni foil, which was used as a fixed input parameter in the data fitting to allow the refinement in the coordination number of the absorption element. In this work, the analysis of the data was performed with the assumption of single scattering with the errors estimated by R-factor. The data fitting is performed in the R-space with k^2-weighted data along with the background. K-range of the data fittingis 2-13 $Å^{-1}$ for **Cs_4K-1** and **Cs_4KH-2** and 2-12 $Å^{-1}$ for PW_9Ni_3/MIL-101(Cr) composite. The R-range of data fitting is 1-4 Å.

X-ray Photoelectron Spectroscopy (XPS) analysis

X-ray photoelectron spectroscopy (XPS) were carried out on the Thermo Scientific model Nexsa G2 X-Ray Photoelectron Spectrometer. The aluminum anode tube for the X-ray emission was operated at a voltage of 12 kV and kept constant during all measurements. Survey scans were obtained at a pass energy of 200 eV, 5 scans with step size 1 eV, whereas for those detailed spectra 40 eV pass energy, 10 scans with 0.1 eV step size were used.

Electron microscopy

Conventional transmission electron microscopy (CTEM) and scanning transmission electron microscopy (STEM) images of the samples were captured on JEM-2100 (JEOL, Japan) electron microscope with a 200kV accelerating voltage. By combining dark-field-STEM with energy-dispersive X-ray spectroscopy (EDS), the elemental spectrum and mapping can be recorded with an Oxford instrument 80mm thin window EDS detector. Statistical analysis of compositions was obtained from elemental maps recorded over regions of several tens of 250 nm to 450 nm in size for arbitrary grain orientations, the data collection time about 600 to 1200 seconds to ensure the EDS minimum integral signal of zero peak is higher than 0.1 cps/eV.

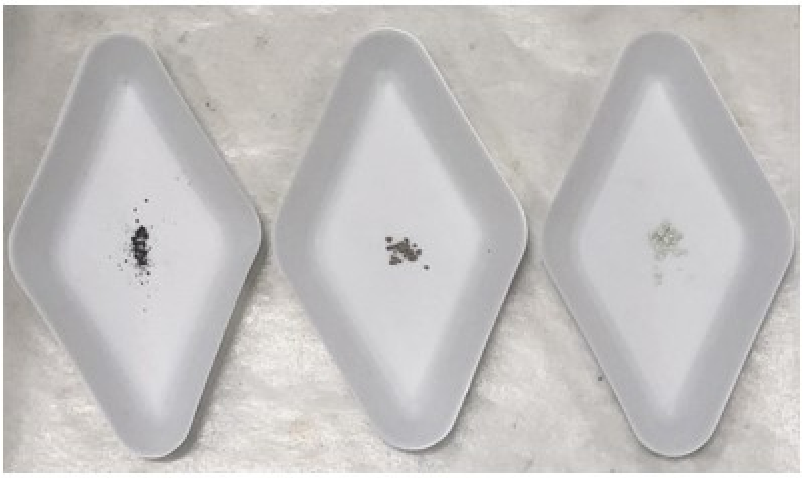

Fig.S1 Photos of freshly prepared (left) and partially reduced (middle) **Cs_4K-1** solid and **Cs_4KH-2** sample (right).

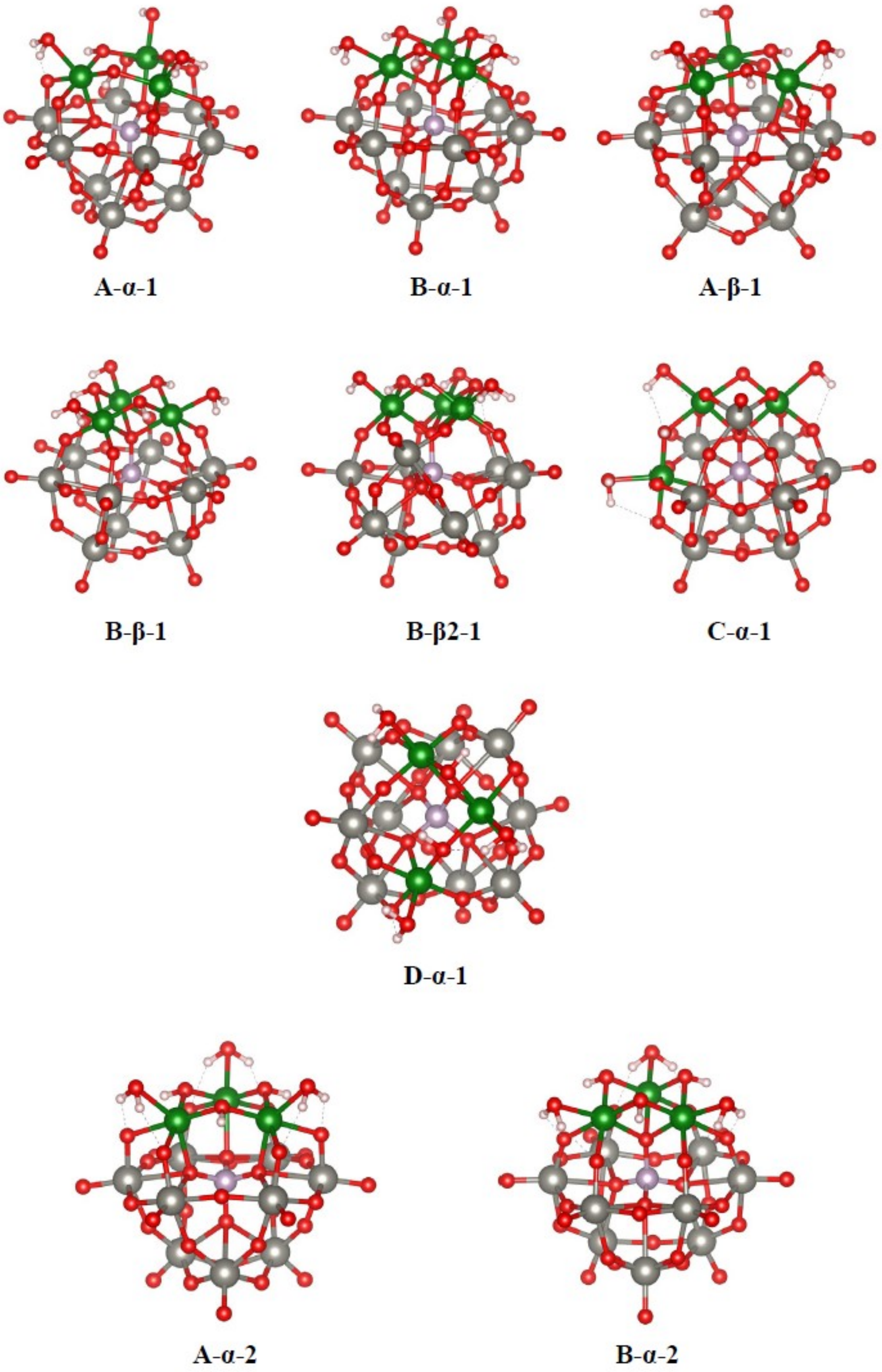

Fig.S2 Selected structure models of $[PW_9Ni^{III}_2Ni^{II}O_{34}(OH)_4(OH_2)_2]^{5-}$ used for computational investigations. Structures with other protonation states were modified based on the structures shown here. See Supporting Information for structural details. P, W, Ni, O and H atoms are shown as purple, grey, green, red and white spheres, respectively.

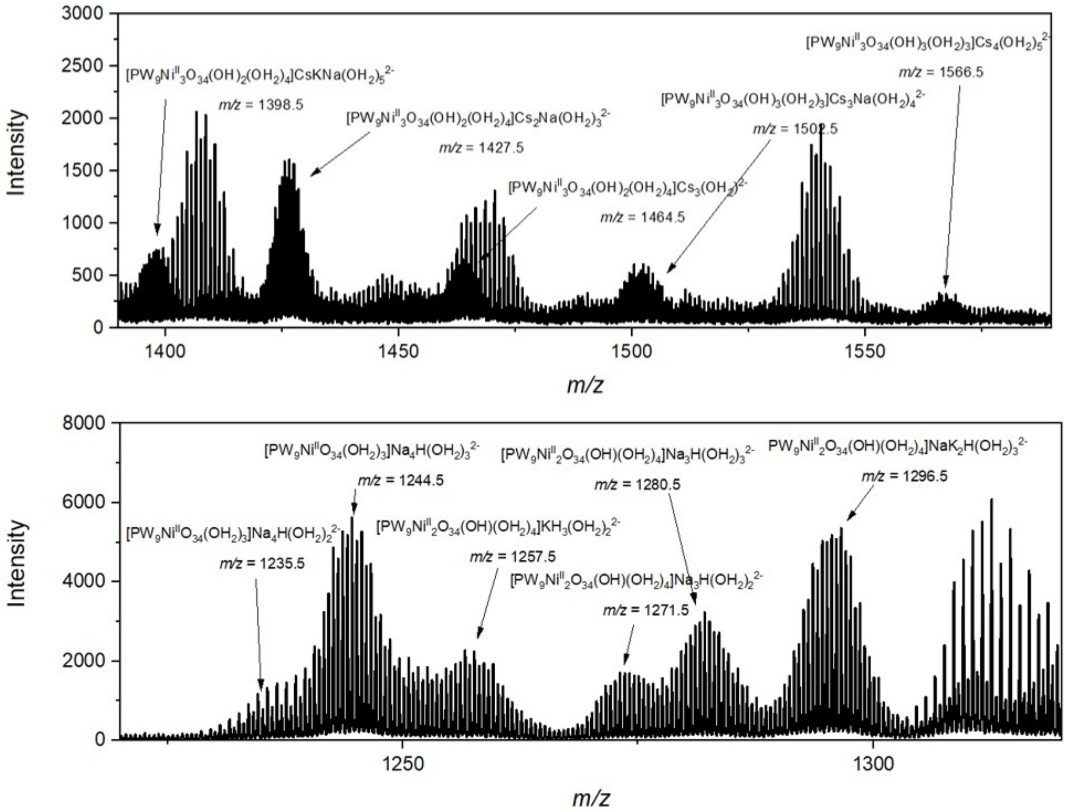

Fig.S3 ESI mass spectrum of **Cs_4KH-2** (above) and corresponding ions with less Ni centres (below) in aqueous solution.

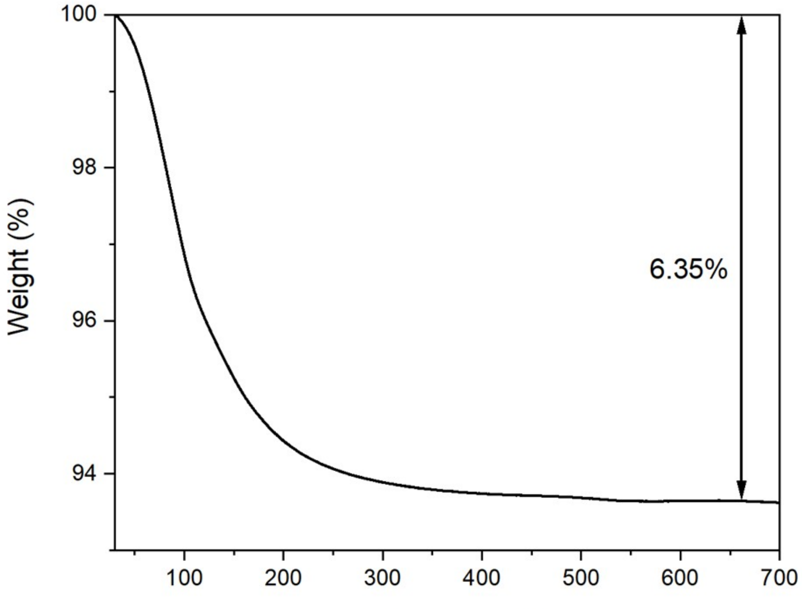

Fig.S4 Thermogravimetric curve of **Cs_4K-1**.

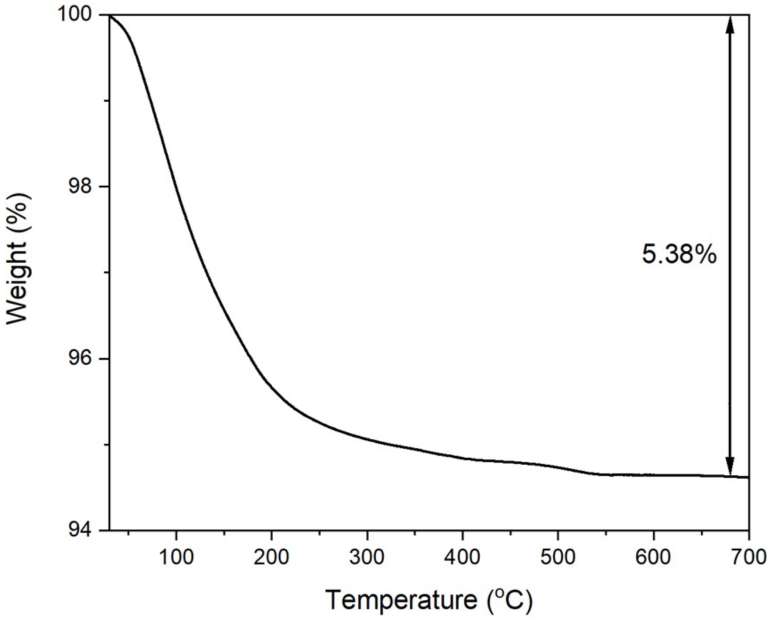

Fig.S5 Thermogravimetric curve of **Cs_4KH-2**.

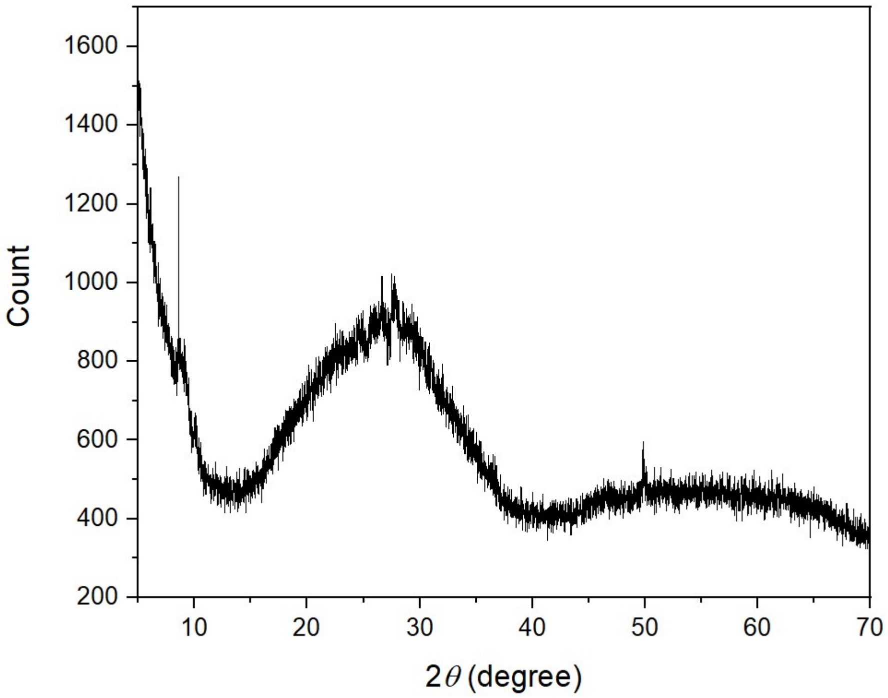

Fig.S6 Powder XRD pattern of **Cs_4K-1**.

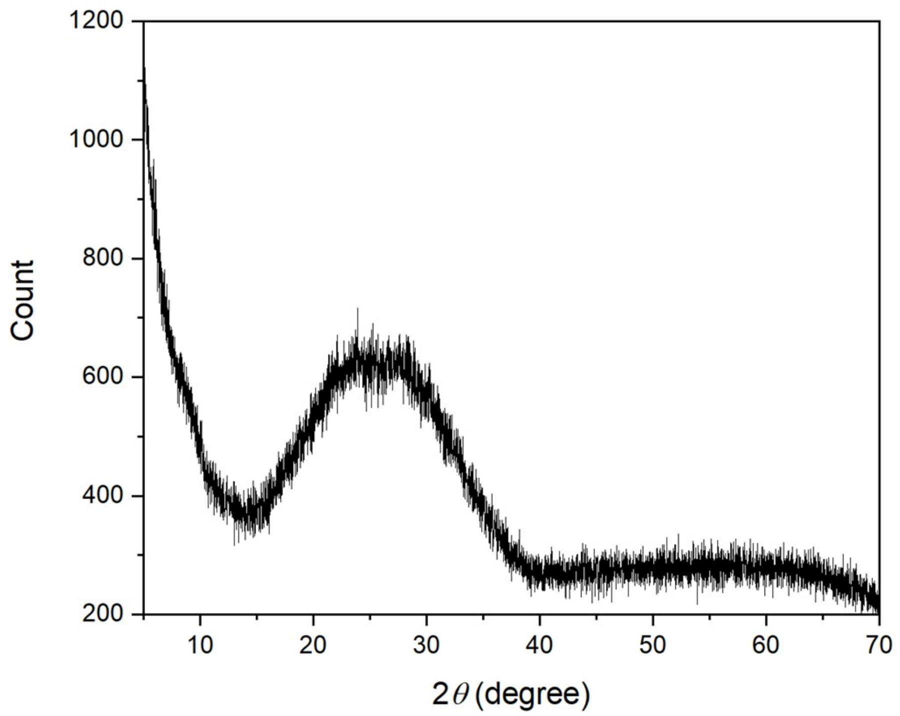

Fig.S7 Powder XRD pattern of **Cs_4KH-2**.

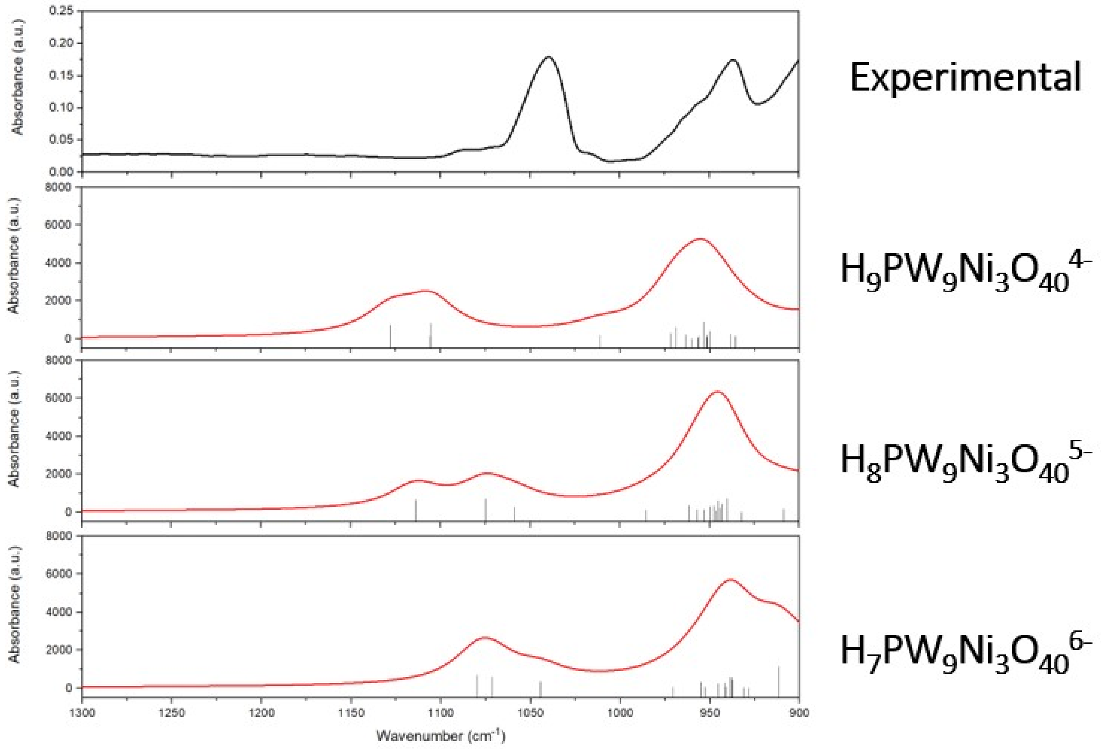

Fig.S8 Comparison of experimental and simulated IR spectra of B-Keggin-type isomers of anion **1** with different protonation states under PBE0-D3BJ/def2svp level.

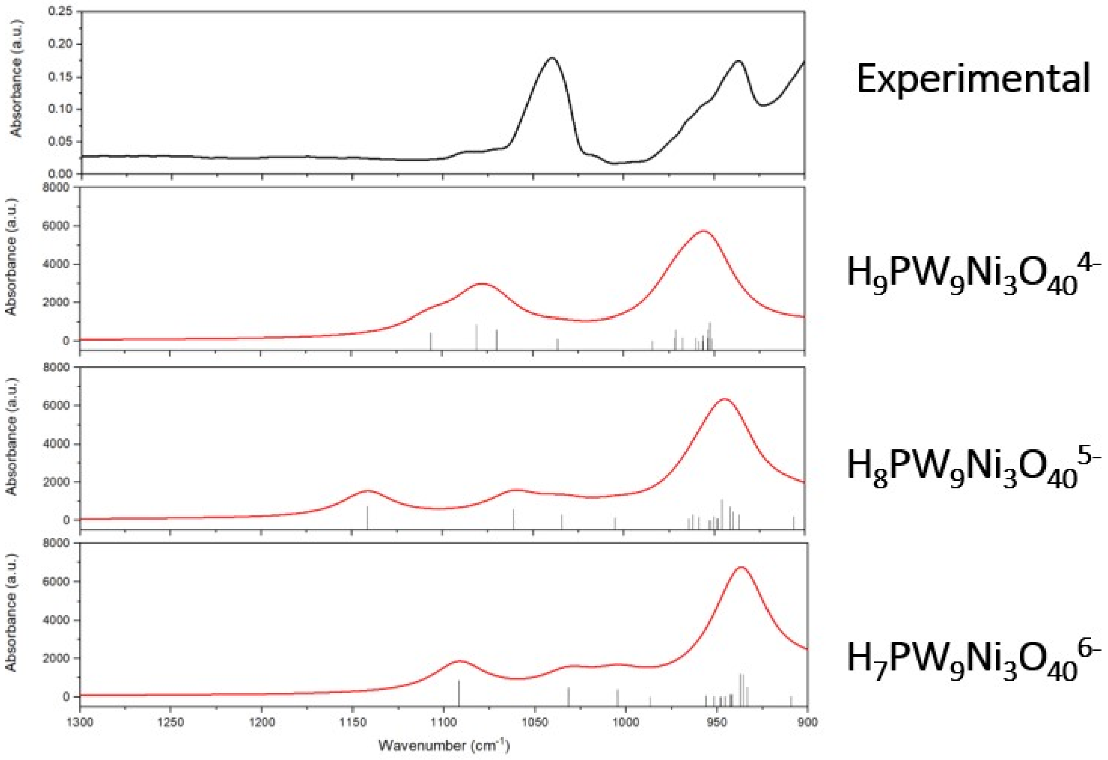

Fig.S9 Comparison of experimental and simulated IR spectra of A-Keggin-type isomers of anion **1** with different protonation states under PBE0-D3BJ/def2svp level.

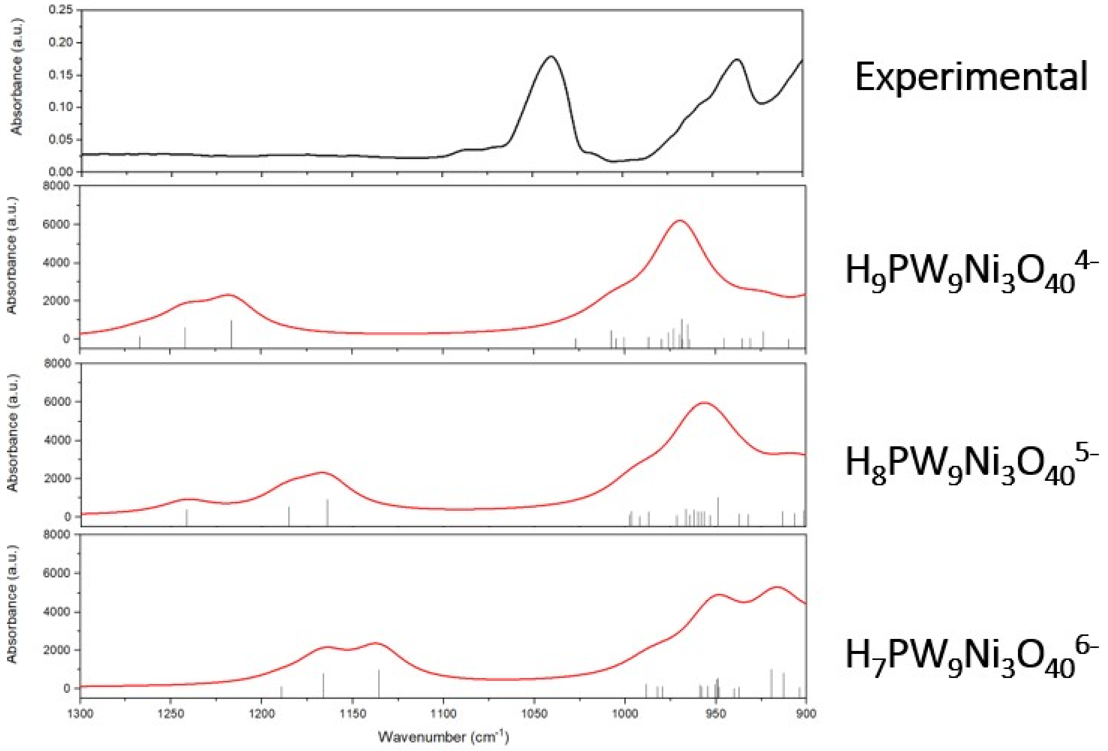

Fig.S10 Comparison of experimental and simulated IR spectra of B-Keggin-type isomers of anion **1** with different protonation states under

PBE0-D3BJ/6-31G(d)/lanl2dz level.

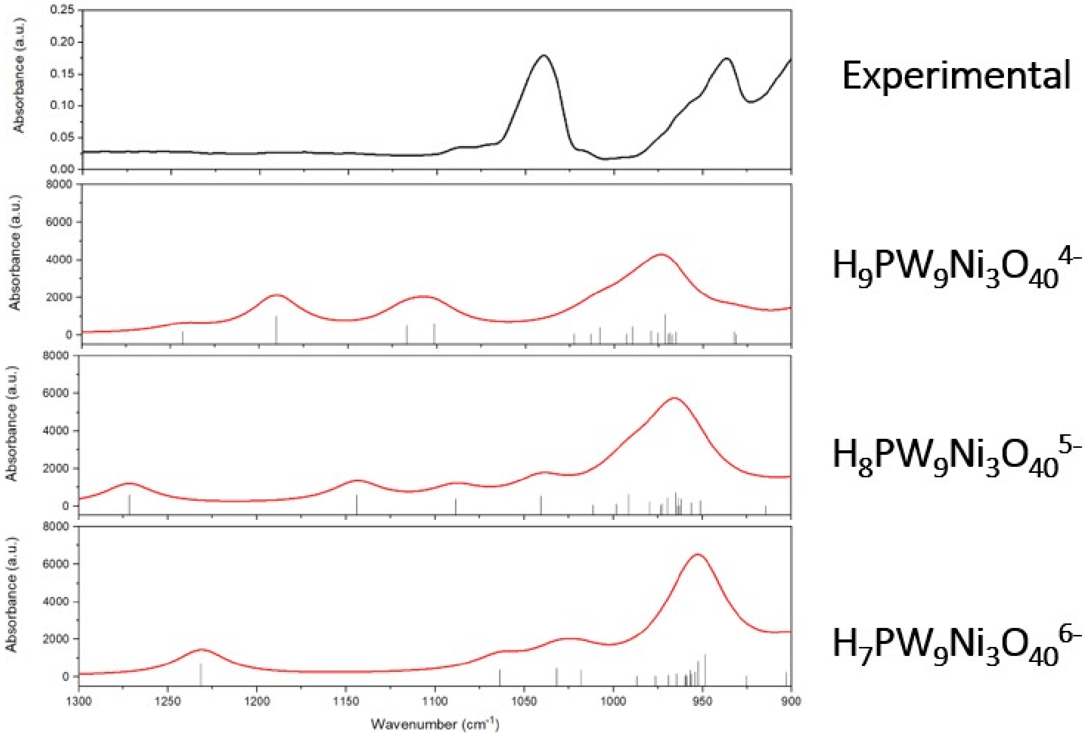

Fig.S11 Comparison of experimental and simulated IR spectra of B-Keggin-type isomers of anion **1** with different protonation states under PBE0-D3BJ/6-31G(d)/lanl2dz level.

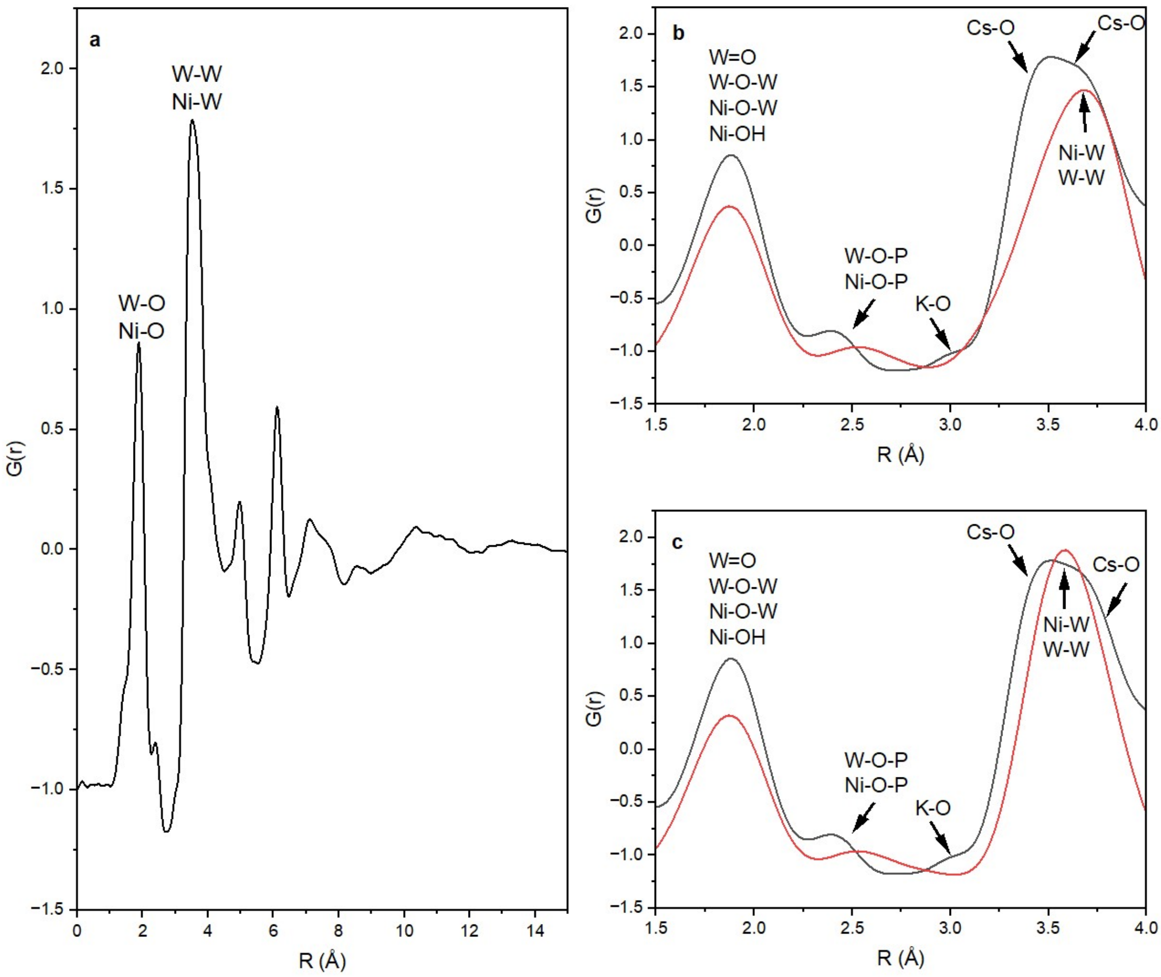

Fig.S12 Pair distribution function analysis of the X-ray total scattering pattern of **Cs_4K-1** (**a**), and its comparison (black line) with simulated (red line) Pair Distribution Function analysis of the X-Ray scattering pattern corresponding to **A-α-1**(b) and **B-α-1**(c).

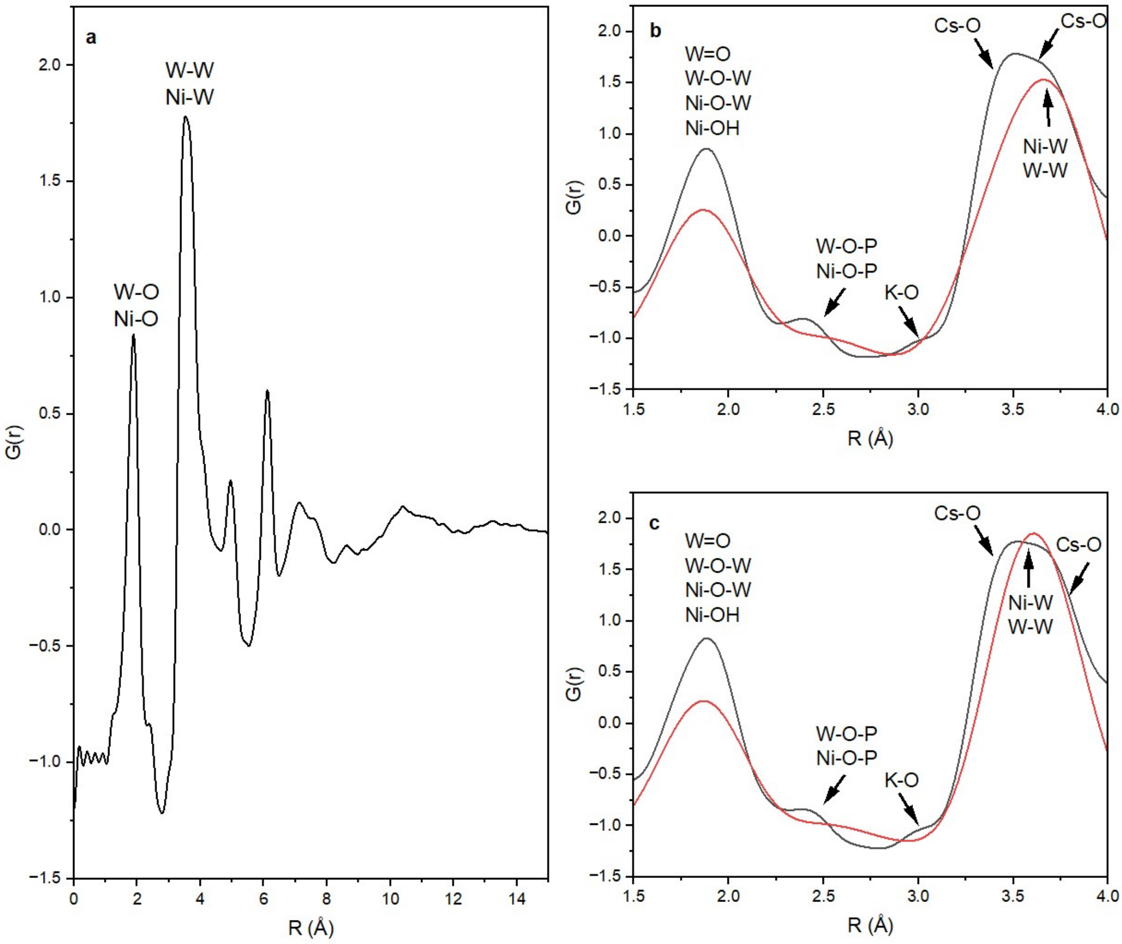

Fig.S13 Pair distribution function analysis of the X-ray total scattering pattern of **Cs_4KH-2** (**a**), and its comparison (black line) with simulated (red line) Pair Distribution Function analysis of the X-Ray scattering pattern corresponding to **A-α-2**(b) and **B-α-2**(c).

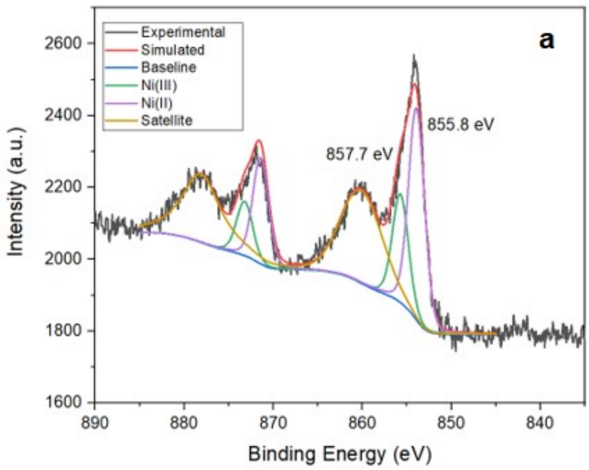

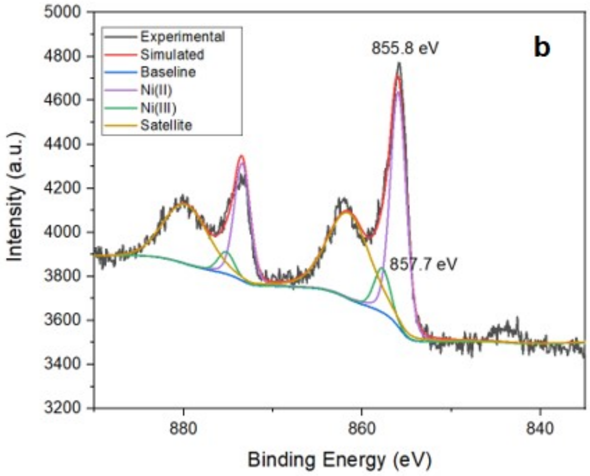

Fig.S14 XPS spectrum of **Cs_4K-1**(a) and **Cs_4KH-2**(b). Small amounts of Cs4K-1 existed in the **Cs_4KH-2** sample due to unavoidable incomplete reduction.

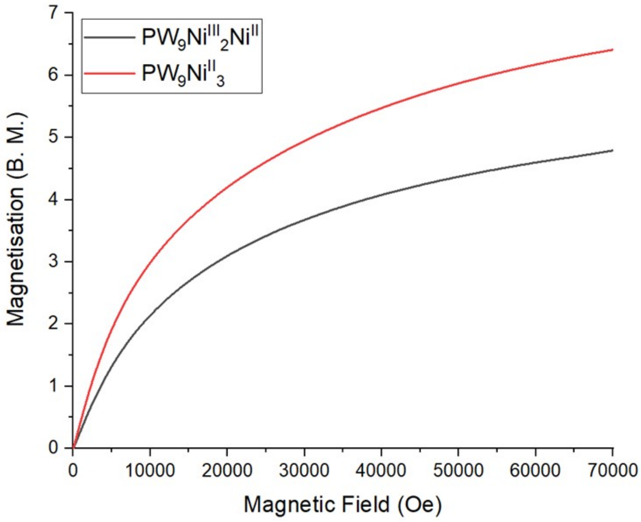

Fig.S15 Magnetisation curve of **Cs_4K-1** and **Cs_4KH-2** samples under different external magnetic fields.

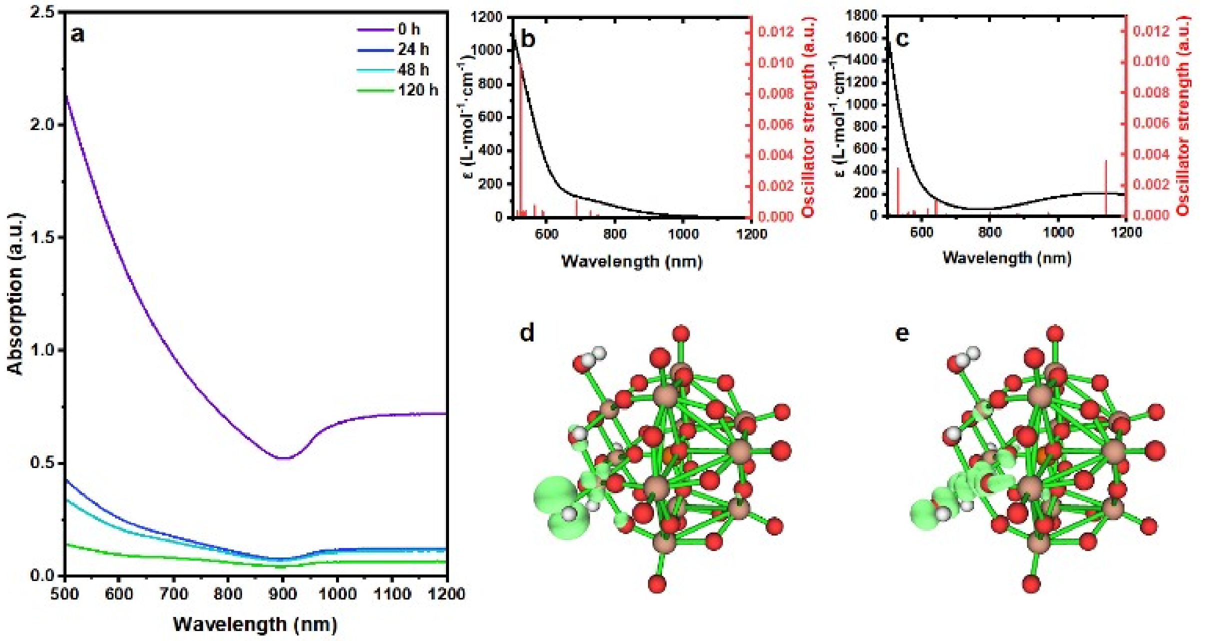

Fig.S16 Time-dependent UV-Vis spectra of **Cs_4K-1** in aqueous solution in the range of 500~1200 nm (a), TD-DFT predicted UV-Vis spectrum (black line) and transitions (red lines) of $[PW_9Ni_3O_{34}(OH)_3(OH_2)_3]^{4-}$ (b) and anion **1** (c), predicted density distribution of holes (d) and photoelectrons (e) for the NIR transition of anion **1**. An isosurface value of 0.002 was used for illustration.

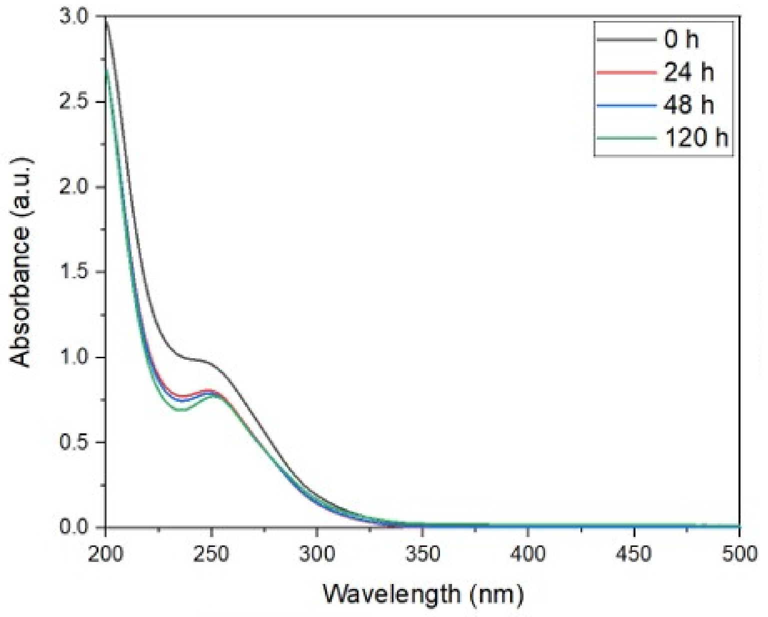

Fig.S17 Time-dependent UV-Vis spectra of Cs_4K-1 in aqueous solution in the range of 200~500 nm.

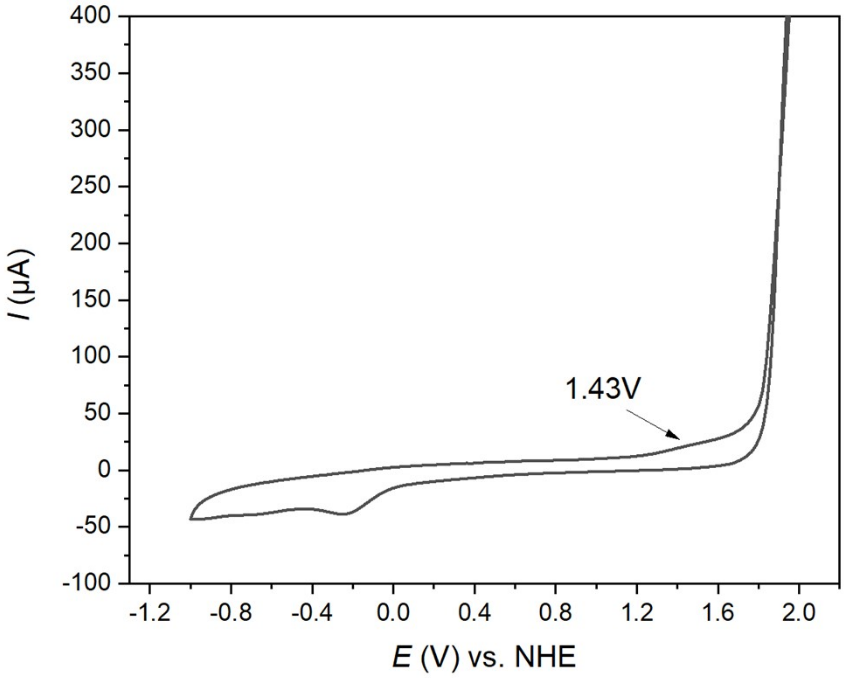

Fig.S18 Overall cyclic voltammogram of **Cs_4K-1** in 0.5M Na_2SO4 solution. The potential value is relative to the Ag/AgCl reference electrode. Redox peaks in the potential range of -1.2 ~ 0V vs. Ag/AgCl corresponds to reduction peaks of oxygen.

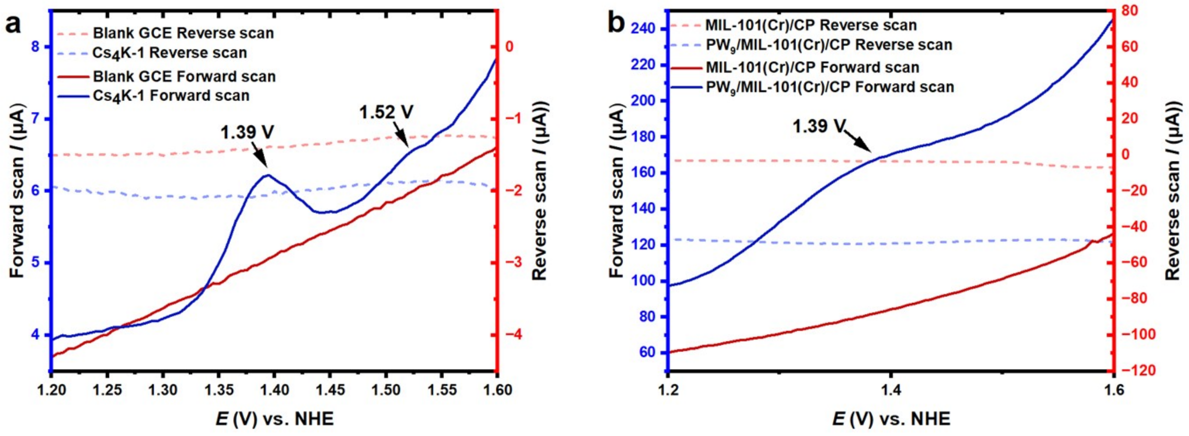

Fig.S19 Differential pulse voltammogram of **Cs_4K-1** (a) and PW_9Ni_3/MIL-101(Cr) composite (b). Forward and reverse scans represent anodic and cathodic currents, respectively.

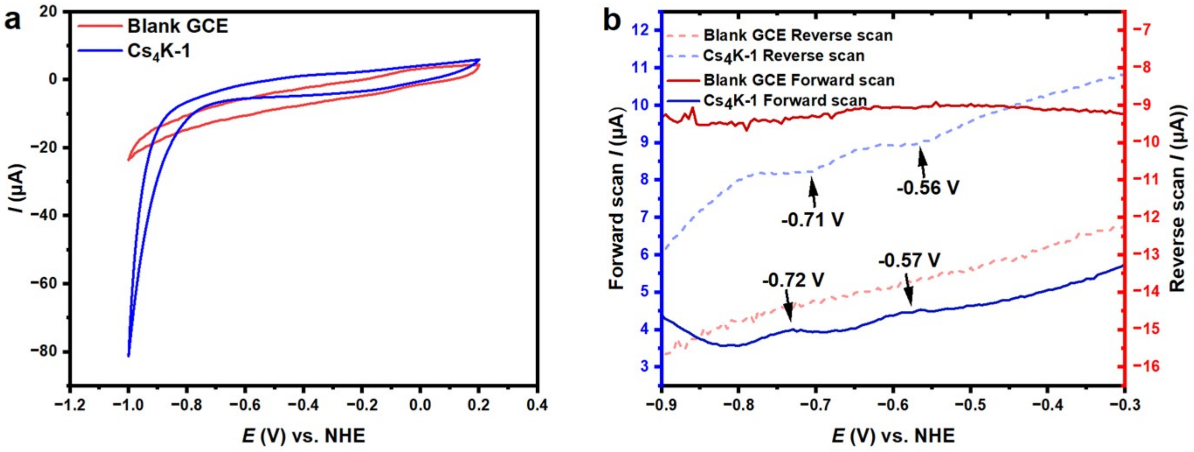

Fig.S20 (a) Cyclic voltammogram of **Cs_4K-1** and blank glassy carbon electrode (GCE) in 0.5M Na_2SO4 solution in the range of -1.0 ~ 0.2V vs. NHE under nitrogen atmosphere. (b) Differential pulse voltammogram and blank glassy carbon electrode (GCE) in 0.5M Na_2SO4 solution in the range of -0.9 ~ 0.3V vs. NHE under nitrogen atmosphere. The peaks corresponding to W^{6+}/W^{5+} redox reactions are marked with arrows. Forward and reverse scan corresponds to anodic and cathodic currents, respectively.

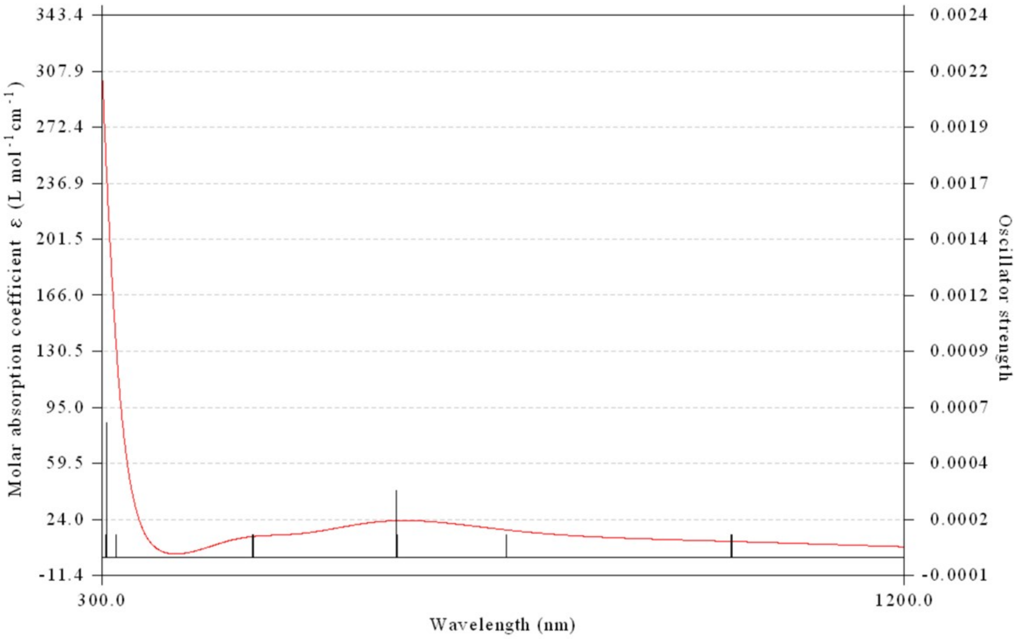

Fig.S21 TD-DFT predicted UV-Vis spectrum (red line) and transitions (black lines) of $[PW_9Ni_3O_{34}(OH)_3(OH_2)_3]^{6-}$.

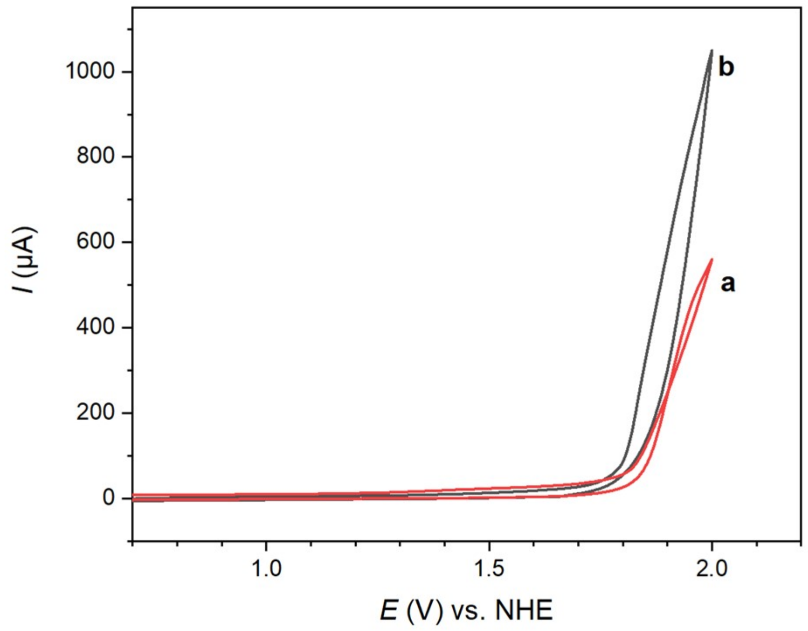

Fig.S22 Comparison of cyclic voltammogram of $\mathbf{Cs_4K}$**-1** in 0.5M Na_2SO4 solution in the range of 0.7 ~ 2.0V vs. NHE before (**a**) and after (**b**) chronoamperometric test.

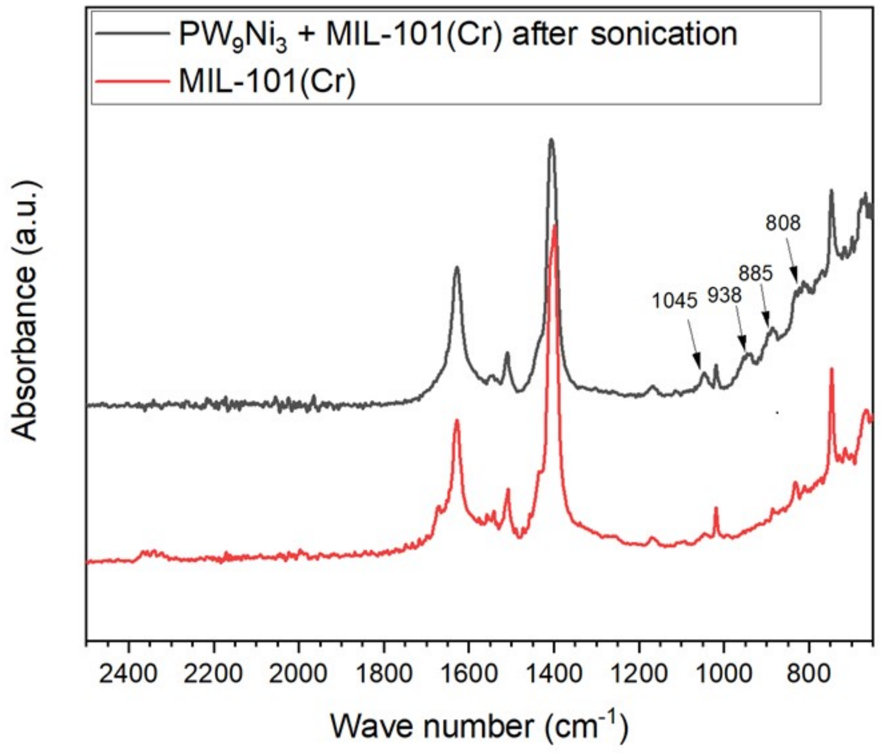

Fig.S23 Comparison of IR spectrua of MIL-101(Cr) and the composite after 20 mins of sonication. Peaks corresponding to anion **1** are indicated by arrows.

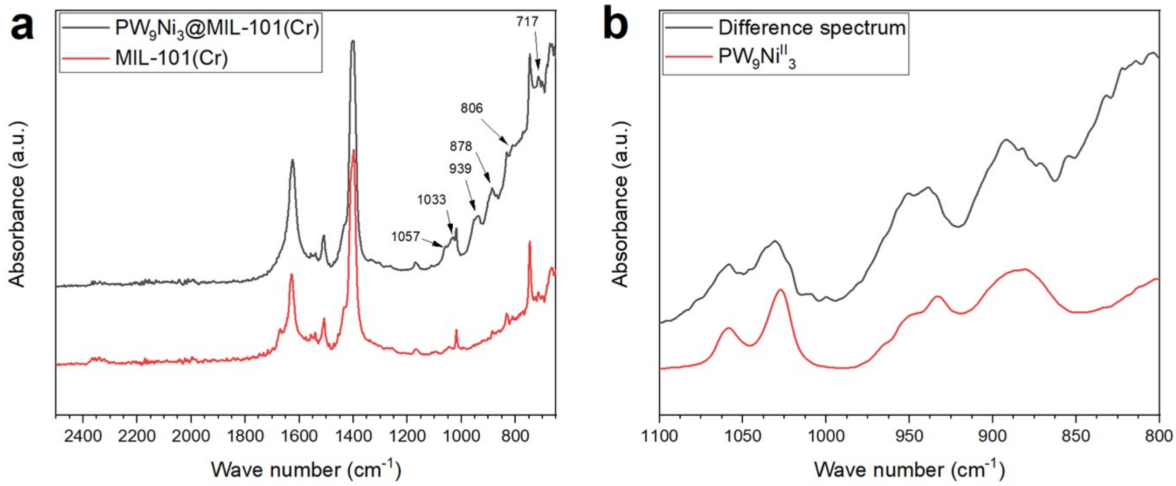

Fig.S24 (**a**) Comparison of IR spectrua of MIL-101(Cr) and the composite. Peaks corresponding to anion **2** are indicated by arrows. (**b**) Comparison of IR spectrua of $\mathbf{Cs_4KH\text{-}2}$ and the difference spectrum between the composite and MIL-101(Cr).

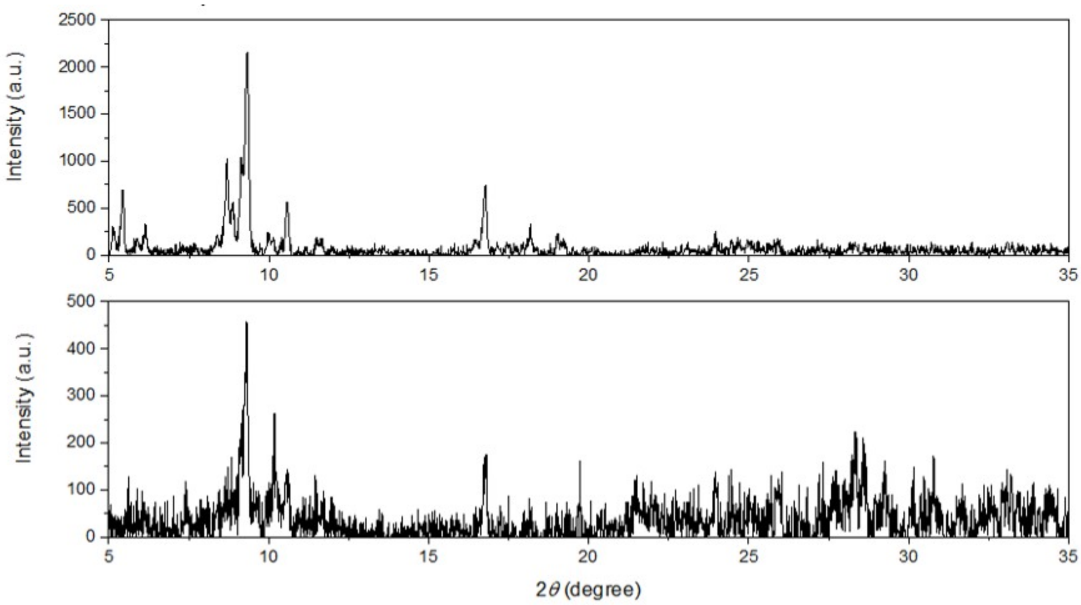

Fig.25 Comparison of XRD patterns between MIL-101(Cr) (above) and polyoxometalate-MOF composite (below)

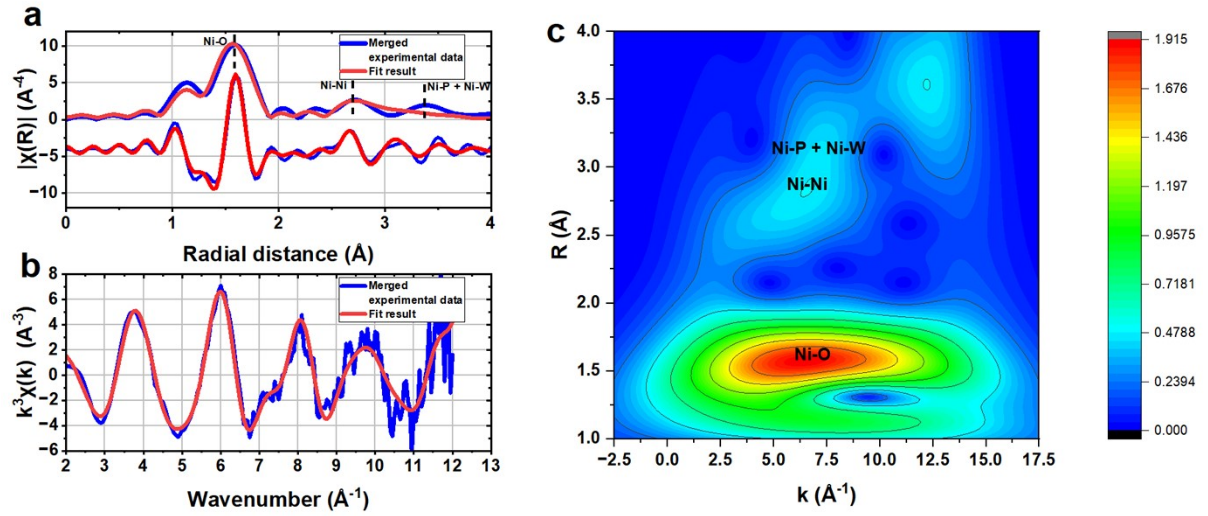

Fig.S26 (a) Comparison of experimental (blue) and simulated (red) k^3-weighted phase shift-corrected Fourier transformed EXAFS result of PW_9Ni_3/MIL-101(Cr) composite at Ni K edge, including the magnitude (above) and real part (below). (b) Comparison of experimental (blue) and simulated (red) EXAFS chi functions of PW_9Ni_3/MIL-101(Cr) composite in k space at Ni K edge. (c) Comparison of experimental (blue) and simulated (red) EXAFS result of PW_9Ni_3/MIL-101(Cr) composite at Ni K edge.

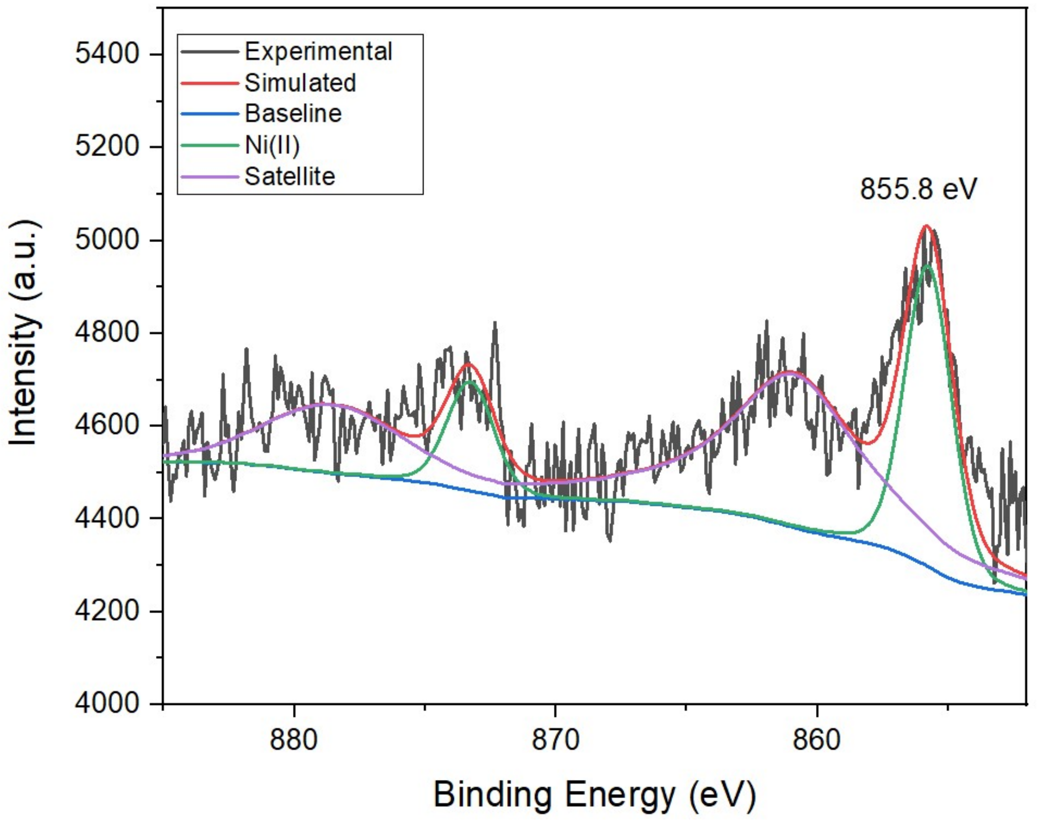

Fig.S27 XPS spectrum of Ni in PW_9Ni_3/MIL-101(Cr) composite.

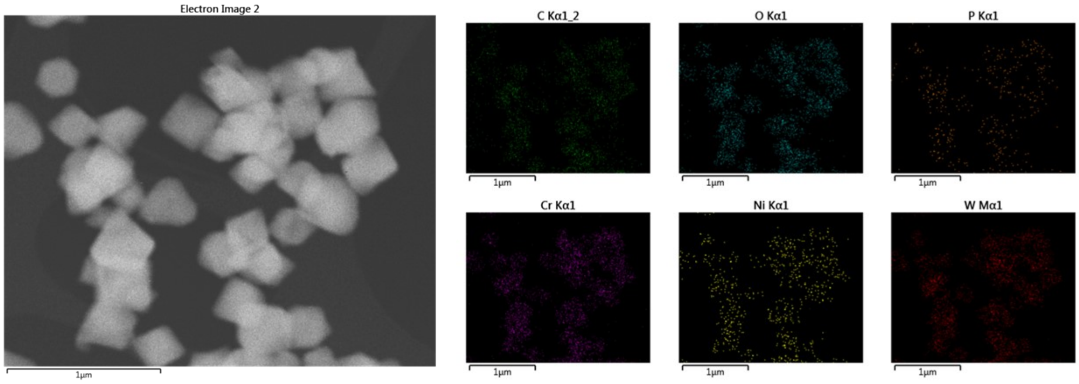

Fig.S28 EDX mapping result of PW_9Ni_3/MIL-101(Cr) composite with multiple crystallites.

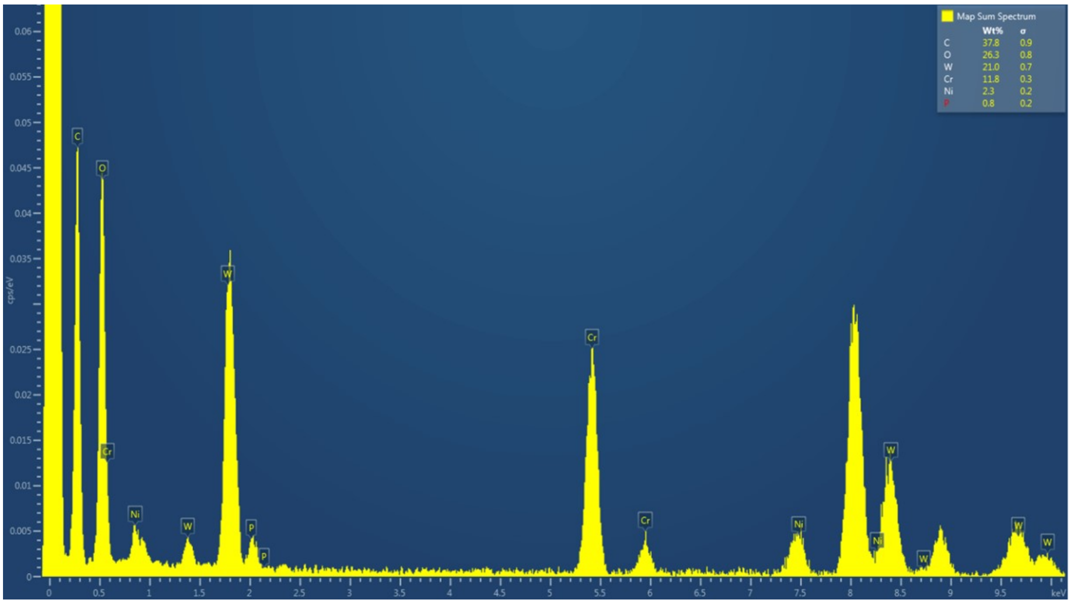

Fig.S29 EDX spectrum of PW_9Ni_3/MIL-101(Cr) composite.

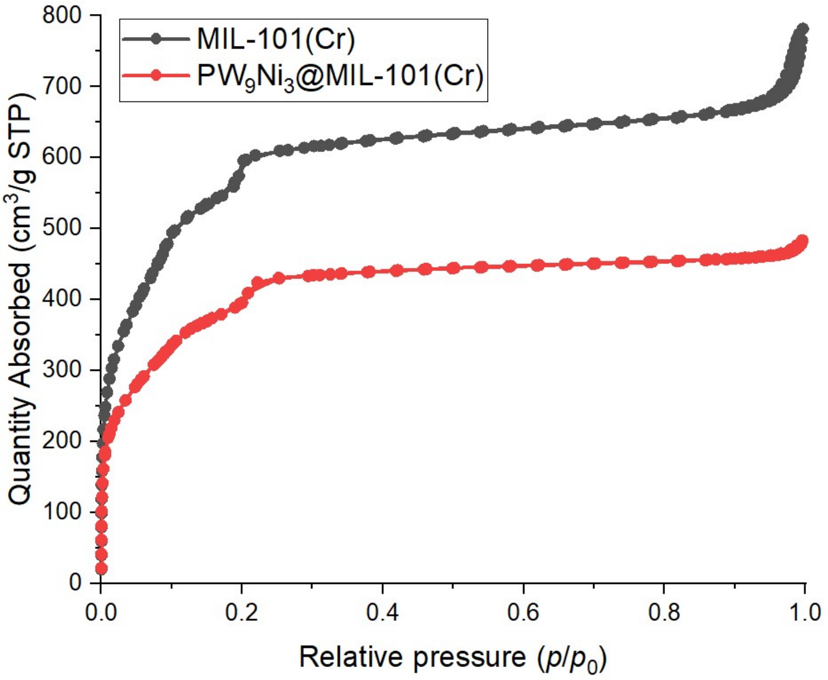

Fig.S30 Brunauer-Emmett-Teller (BET) curves of MIL-101(Cr) and the composite.

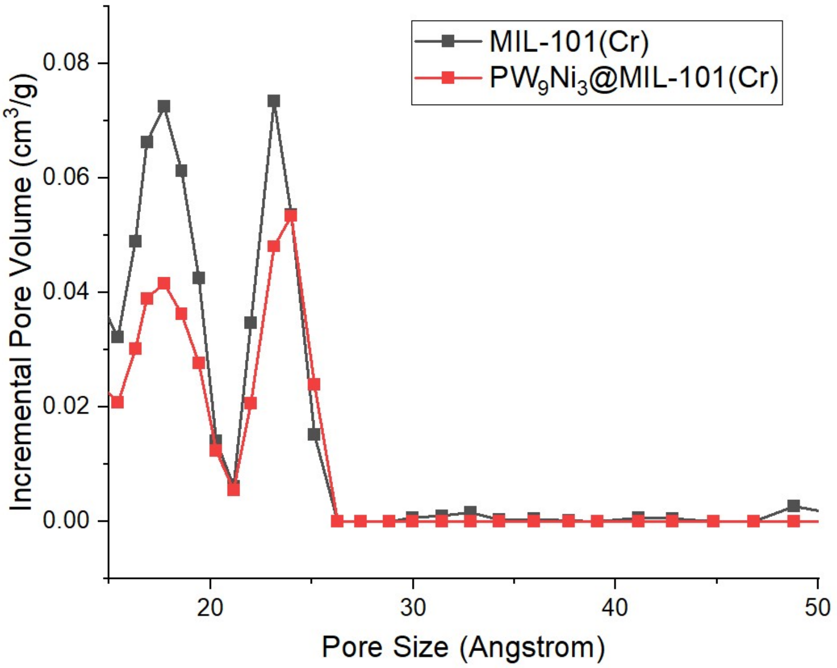

Fig.S31 Pore distribution of the PW_9Ni_3/MIL-101(Cr) composite.

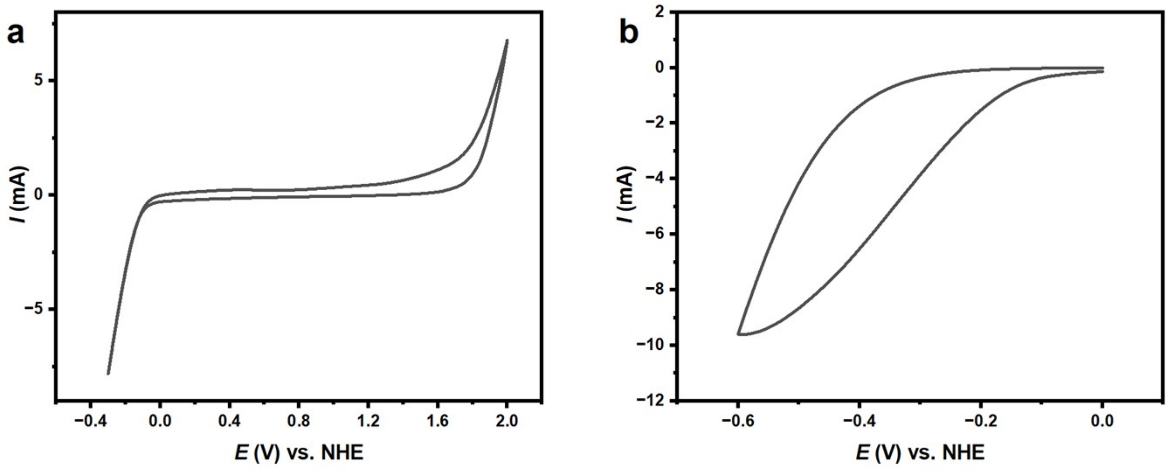

Fig.S32 (a) Overall cyclic voltammogram of PW_9Ni_3/MIL-101(Cr) composite in 0.5M Na_2SO4 solution in the range of -0.3 ~2.0 V vs. NHE. The strong current occurred in the range of $E < 0$ V corresponds to the ORR current. (b) Cyclic voltammogram of PW_9Ni_3/MIL-101(Cr) composite in 0.5M Na_2SO4 solution in the range of -0.6 ~0 V vs. NHE.

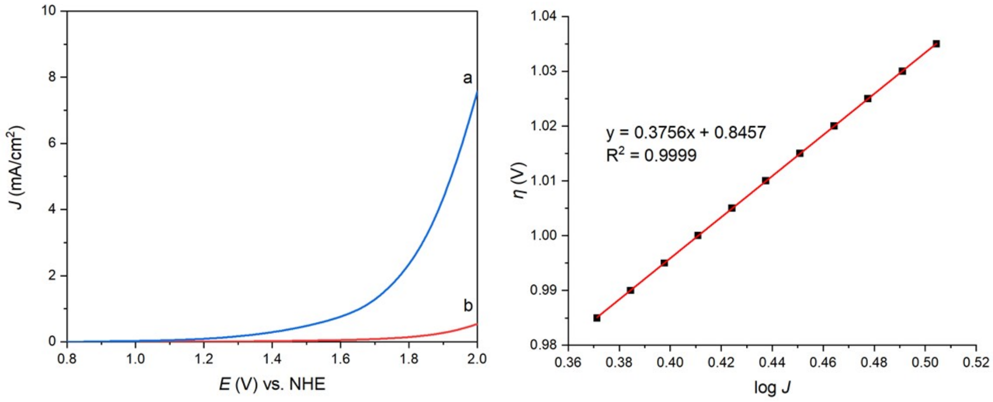

Fig.S33 Left: linear scan voltammogram of PW_9Ni_3/MIL-101(Cr) composite (a) and MIL-101(Cr) (b) on carbon paper. Right: Tafel plots of PW_9Ni_3/MIL-101(Cr) composite based on LSV data.

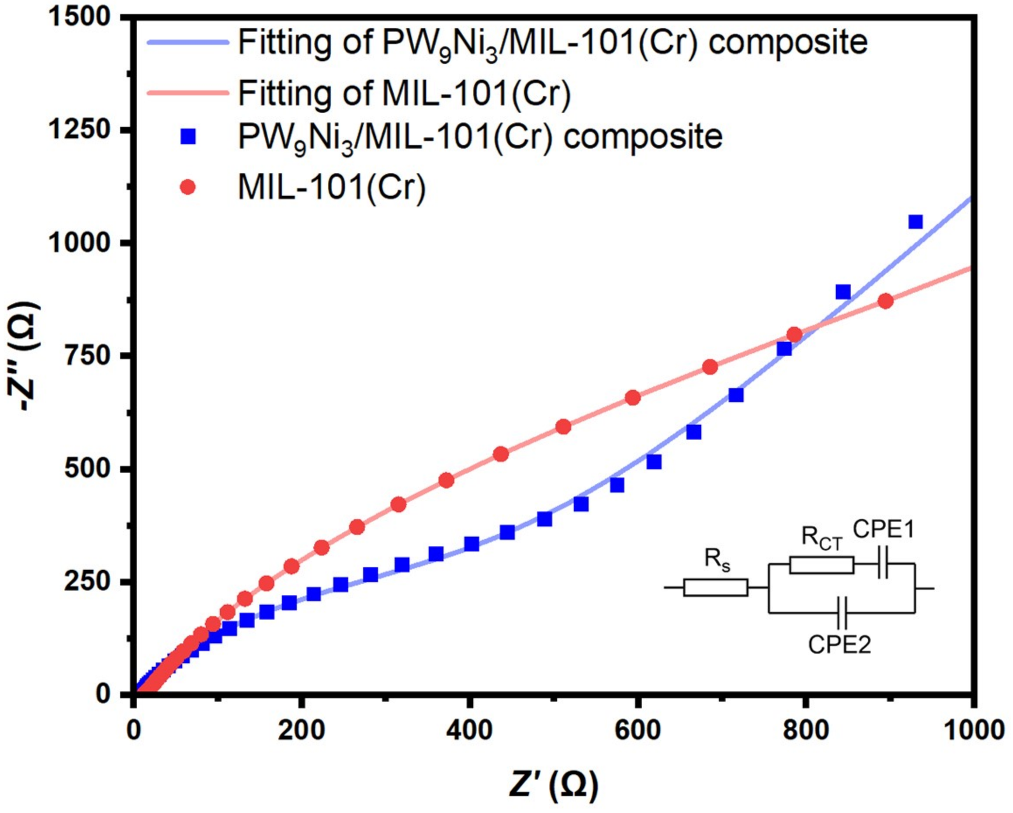

Fig.S34 Electrochemical impedance plots of MIL-101(Cr) and PW_9Ni_3/MIL-101(Cr) composite on carbon paper electrode, showing the proposed equivalent circuit used for fitting.

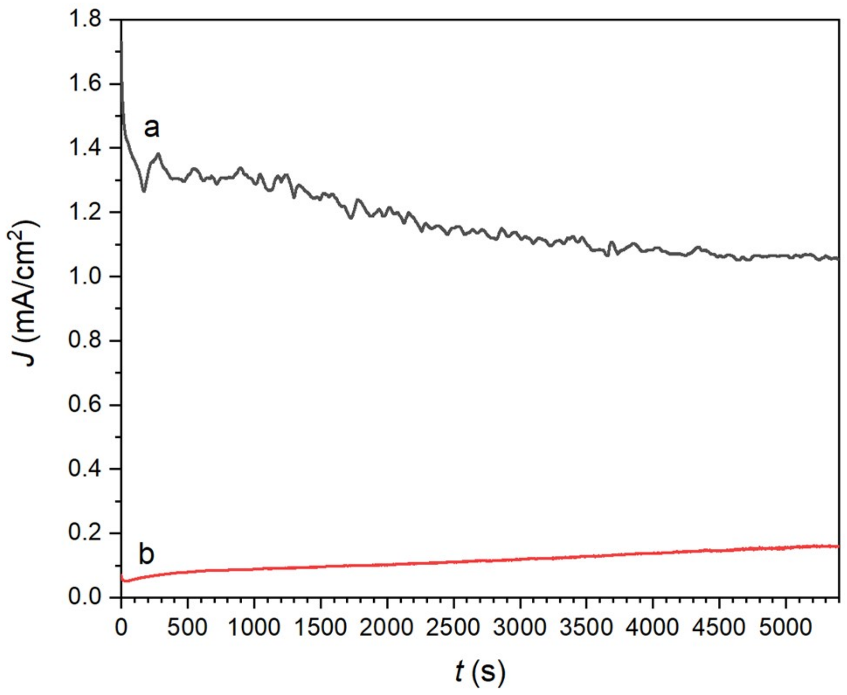

Fig.S35 Chronoamperometric test of the PW_9Ni_3/MIL-101(Cr) composite and raw MIL-101(Cr) loaded on carbon paper electrodes under applied potential of 1.65V vs. Ag/AgCl.

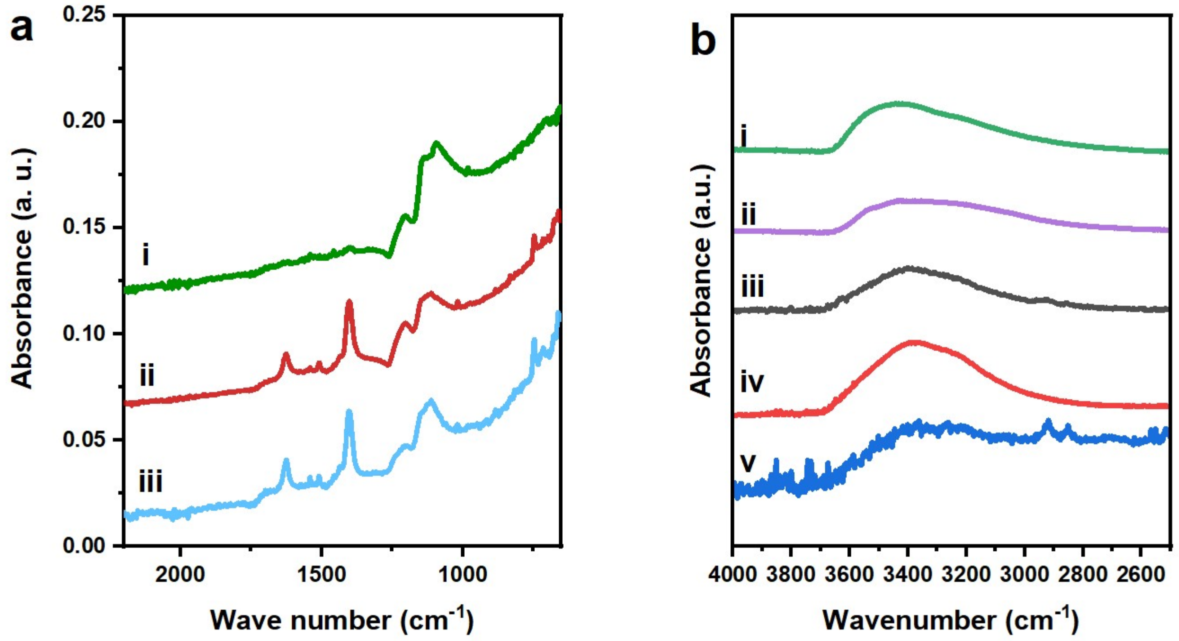

Fig.S36 (a) Comparison of IR spectra of (i) raw carbon paper, (ii) MIL-101(Cr) and (iii) PW_9Ni_3/MIL-101(Cr) composite after electrolytic test. (b) Comparison of IR spectra of (i) Cs_4K-1, (ii) Cs_4KH-2, (iii) PW_9Ni_3/MIL-101(Cr) composite after 20 mins of sonication, (iv) PW_9Ni_3/MIL-101(Cr) composite and (v) PW_9Ni_3/MIL-101(Cr) composite after catalytic test, in the wavenumber range of 2500~4000 cm^{-1}.

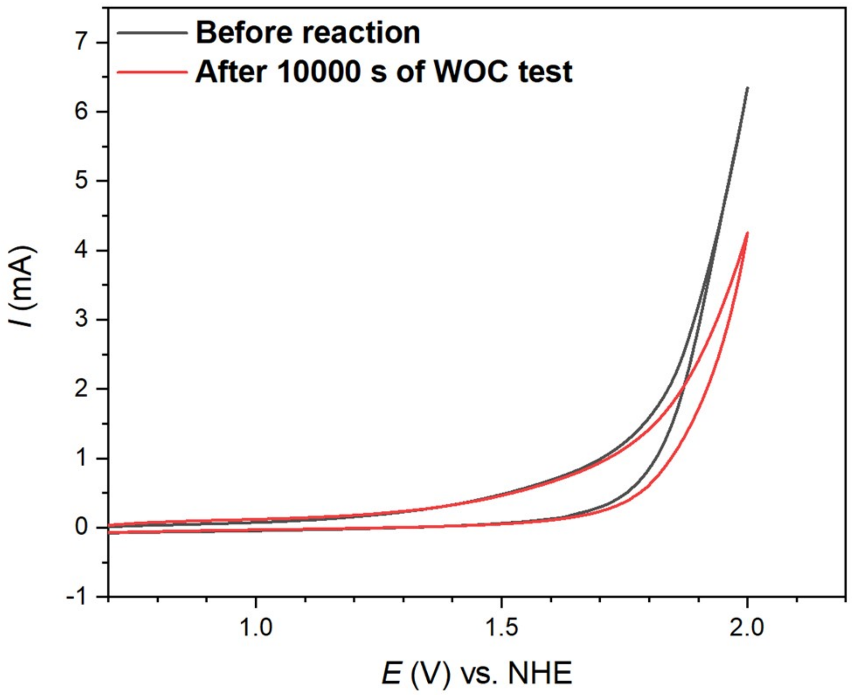

Fig.S37 Cyclic voltammogram of the PW_9Ni_3/MIL-101(Cr) composite before and after 10000 s of OER test.

Table S1. Comparison of simulated and experimental ESI-MS peaks centered at m/z=1360.6.

Experimental m/z	Relative Intensity	Simulated m/z	Relative Intensity
1355.5728	0.1821	1355.5631	0.1783
1356.0647	0.2570	1356.0592	0.2509
1356.5568	0.3369	1356.5607	0.3601
1357.0724	0.4457	1357.0621	0.4558
1357.5646	0.5737	1357.5636	0.5954
1358.0569	0.6387	1358.0651	0.6834
1358.5728	0.7886	1358.5611	0.8244
1359.0653	0.8642	1359.0626	0.8704
1359.5579	0.8917	1359.5641	0.9821
1360.0741	0.9732	1360.0655	0.9463
1360.5669	1.0000	1360.5671	1
1361.0797	0.8733	1361.0686	0.8786
1361.5762	0.8990	1361.5646	0.8837
1362.0692	0.7742	1362.0661	0.7127
1362.5624	0.6738	1362.5676	0.6832
1363.0791	0.5699	1363.0691	0.4975
1363.5724	0.5065	1363.5706	0.4581
1364.0659	0.3553	1364.0667	0.3007
1364.5829	0.3204	1364.5681	0.2714
1365.0765	0.2197	1365.0691	0.158
1365.5702	0.1843	1365.5711	0.1402

Table S2. Comparison of simulated and experimental ESI-MS peaks centered at m/z=1426.5.

Experimental m/z	Relative Intensity	Simulated m/z	Relative Intensity
1421.5242	0.1924	1421.5096	0.1791
1422.0040	0.2569	1422.0074	0.25
1422.5079	0.3470	1422.5108	0.3594
1423.0119	0.4240	1423.0085	0.4529
1423.5159	0.5584	1423.5119	0.5948
1424.0201	0.6615	1424.0096	0.6818
1424.5243	0.7456	1424.5130	0.827
1425.0046	0.8429	1425.0106	0.8618
1425.5090	0.9147	1425.5140	0.9802
1426.0135	0.9237	1426.0118	0.9416
1426.5181	1.0000	1426.5159	1
1427.0228	0.8925	1427.0129	0.8723
1427.5276	0.8763	1427.5163	0.8844
1428.0324	0.7250	1428.0141	0.7103
1428.5133	0.7226	1428.5174	0.6814
1429.0183	0.5712	1429.0151	0.4967

Experimental m/z	Relative Intensity	Simulated m/z	Relative Intensity
1429.5235	0.4900	1429.5185	0.4589
1430.0287	0.3779	1430.0162	0.3009
1430.5340	0.3179	1430.5196	0.2706
1431.0153	0.2253	1431.0173	0.1578
1431.5208	0.1969	1431.5207	0.1389

Table S3. Computated relative electronic energy (Δ*E*) and Gibbs free energy (ΔG_{298K}) for selected structures compared with structure B-α-1.

Structure	***E*** (kcal/mol)	***G*** (kcal/mol)
**A-α-1**	2.3	1.3
**B-α-1**	0.0	0.0
**A-β-1**	6.6	6.0
**B-β-1**	3.3	3.0
**B-β2-1**	7.4	7.0
**C-α-1**	4.2	5.3
**D-α-1**	6.2	7.0

Table S4. Computated relative electronic energy (Δ*E*) and Gibbs free energy (ΔG_{298K}) of isomer A-α-2 and B-α-2.

Structure	***E*** (kcal/mol)	***G*** (kcal/mol)
**A-α-2**	3.1	2.8
**B-α-2**	0.0	0.0

Table S5. Cartesian structural coordinates of optimized structure A-α-1.

Atom	x	y	z
W	0.995161	1.777294	2.611960
W	0.838261	-1.706150	2.683035
W	-2.107440	0.158364	2.549983
W	0.070781	3.584134	-0.554380
W	3.252628	1.581267	-0.370880
W	3.099554	-1.881460	-0.278530
W	-0.246250	-3.614580	-0.389390
W	-3.160970	-1.758110	-0.508670
W	-3.025850	2.016686	-0.577880
Ni	-0.826800	1.733375	-2.996160
Ni	-0.961560	-1.699060	-2.903530
Ni	2.146984	-0.172680	-2.800810
P	-0.009810	-0.008160	-0.382340
O	1.620981	2.839409	3.803336
O	1.372183	-2.750220	3.934367
O	-0.056830	0.061402	1.158778
O	-3.339880	0.241840	3.742893
O	-0.202620	3.410690	-2.364320
O	0.388484	5.259390	-0.387710
O	3.347084	1.145788	-2.177210
O	4.624987	2.586647	-0.169630
O	3.223603	-1.561440	-2.104700
O	-0.468700	-3.575100	-2.174580
O	-2.914870	-2.016890	-2.270480
O	-4.761160	-2.322070	-0.250680
O	-2.776440	2.177539	-2.334890
O	-4.572600	2.722342	-0.330060
O	1.476285	-0.087090	-0.826060
O	-0.777330	-1.263990	-0.873370
O	-0.676390	1.261065	-1.005920
O	-1.512150	0.084405	-3.581020
O	0.965313	-1.469970	-3.440910
O	1.098075	1.199111	-3.459640
O	1.446082	0.041955	3.306583
O	-0.770000	1.443665	3.245502
O	0.296267	2.941729	1.290584

Atom	x	y	z
O	2.472535	1.577883	1.404084
O	-0.880170	-1.160350	3.327020
O	2.332348	-1.677890	1.494869
O	0.026576	-2.842760	1.452720
O	-2.696480	-1.149380	1.373366
O	-2.589330	1.455816	1.313270
O	1.843638	2.834821	-0.746650
O	-1.816130	3.491454	-0.212630
O	3.946828	-0.170430	0.048806
O	1.595885	-3.029890	-0.562300
O	-2.119250	-3.326490	-0.051800
O	-3.410070	0.133924	-0.727860
O	4.382559	-2.984320	-0.009100
O	-0.077170	-5.288190	-0.049500
O	-1.078050	2.489069	-4.626580
O	3.593289	-0.278950	-4.435860
O	-1.657580	-2.916380	-4.536490
H	1.382718	-2.288290	-3.130330
H	1.595173	1.986191	-3.186330
H	-2.452160	0.172641	-3.365750
H	-1.281690	-3.694170	-4.087160
H	-0.817520	3.397952	-4.411280
H	4.065299	0.467587	-4.028260
H	3.996537	-1.047320	-3.996650
H	-2.555510	-2.874810	-4.163290

Table S6. Cartesian structural coordinates of optimized structure B-α-1.

Atom	x	y	z
W	-0.691660	0.063387	-3.566600
W	-0.687010	3.063263	-1.818720
W	2.274170	1.216157	-2.118190
W	2.608034	1.205077	1.619202
W	-0.018340	-3.163880	1.742745
W	-0.373610	-3.141020	-1.726240
W	2.608280	-2.024410	-0.257930
W	-0.361340	3.059884	1.874106
W	-0.005560	0.056056	3.608026

Atom	x	y	z
Ni	-3.188970	1.537667	0.282399
Ni	-2.827060	-1.067770	1.857074
Ni	-3.192670	-1.071260	-1.197820
P	-0.391480	-0.018640	0.003724
O	0.200739	-1.446100	-0.026310
O	0.156329	0.747480	1.232868
O	-0.031800	0.746402	-1.292700
O	-1.955000	-0.145960	0.122680
O	1.640607	2.775829	1.929667
O	4.193789	1.629857	2.112999
O	1.861996	0.485032	3.226764
O	-4.261420	0.472445	-1.047950
O	-3.898290	0.462544	1.873234
O	-0.106690	3.389124	0.003587
O	0.411973	-1.698830	2.914209
O	-0.545090	-4.502090	-2.759300
O	1.297454	2.803292	-2.183110
O	-2.314580	2.609123	-1.220070
O	-0.286650	1.950499	3.482865
O	-0.854500	1.994791	-3.415520
O	1.844841	-3.083110	1.150556
O	0.021670	-4.524200	2.787372
O	-0.127210	-1.692040	-2.959600
O	2.829683	-0.628880	1.030183
O	3.759848	1.647989	-2.859390
O	-4.393470	3.297905	0.403520
O	-0.451530	4.639847	2.544337
O	-3.911330	-1.860840	0.582650
O	-2.285250	-0.191190	-2.825360
O	-2.083900	-2.590380	-1.435620
O	-0.336760	-3.986620	0.028799
O	0.037509	-0.182270	5.306420
O	-3.736080	-2.026020	3.571200
O	4.183728	-2.686980	-0.396200
O	-2.077460	2.614344	1.597107
O	2.626240	1.582256	-0.241230
O	1.594454	-3.079930	-1.474810

Atom	x	y	z
O	-1.778710	-2.615970	1.839815
O	2.623028	-0.616080	-1.530260
O	-1.767900	-0.278680	3.177920
O	1.289354	0.503751	-3.534810
O	-0.899400	4.653505	-2.434830
O	-4.425150	-1.877580	-2.252110
O	-0.950190	-0.178430	-5.250910
H	-4.065380	0.893692	-1.898410
H	-3.980380	3.655510	-0.401320
H	-3.897470	-2.622230	-2.579040
H	-3.206420	-2.829880	3.438357
H	-3.820590	3.646124	1.109245
H	-3.490640	0.954751	2.604040
H	-3.513850	-2.744680	0.523081
H	-3.188390	-1.500630	4.178631

Table S7. Cartesian structural coordinates of optimized structure A-β-1.

Atom	x	y	z
W	-1.194710	-1.833810	-2.510860
W	1.868895	-0.173170	-2.738300
W	-1.097070	1.670447	-2.628630
W	-0.200130	-3.576650	0.605814
W	3.150944	-1.788430	0.348148
W	3.244146	1.643997	0.194746
W	0.007636	3.632984	0.335232
W	-3.020370	1.999884	0.543359
W	-3.151790	-1.792620	0.678096
Ni	-0.873640	-1.619820	3.048442
Ni	-0.781130	1.789990	2.900286
Ni	2.190786	0.070963	2.779585
P	-0.004610	0.016199	0.380960
O	-1.857800	-2.918580	-3.662740
O	3.016354	-0.259100	-4.007910
O	-0.077750	-0.070690	-1.157530
O	-1.709790	2.672670	-3.879550
O	-0.287110	-3.318820	2.428194
O	0.036324	-5.267800	0.458451

Atom	x	y	z
O	3.232203	-1.401610	2.162359
O	4.420091	-2.919470	0.132240
O	3.294575	1.433866	2.037478
O	-0.066530	3.594115	2.134689
O	-2.753260	2.146361	2.315586
O	-4.596450	2.641486	0.316117
O	-2.909580	-1.826550	2.441990
O	-4.762730	-2.354560	0.470504
O	1.488179	-0.002840	0.815054
O	-0.672700	1.331331	0.870169
O	-0.753600	-1.189570	1.041261
O	-1.431380	0.069664	3.646731
O	1.120962	1.440461	3.449791
O	1.072451	-1.199190	3.511532
O	0.581992	-1.489820	-3.263730
O	-1.643950	-0.111390	-3.238880
O	-0.317200	-2.975340	-1.256700
O	2.535129	-1.436640	-1.465910
O	0.642001	1.133228	-3.381670
O	2.595638	1.161320	-1.585230
O	-0.168580	2.849091	-1.516090
O	-2.469330	1.639042	-1.376970
O	-2.543630	-1.644860	-1.247590
O	1.619586	-2.911490	0.552731
O	-2.108100	-3.429380	0.515450
O	4.059471	-0.101670	0.064538
O	1.781613	2.868172	0.280635
O	-1.915230	3.557119	0.225467
O	-3.353430	0.112198	0.591315
O	4.573024	2.678942	-0.121610
O	0.316381	5.282992	-0.024020
O	-1.165160	-2.365060	4.677243
O	3.699264	0.094756	4.350722
O	-1.367460	3.113890	4.488494
H	1.587827	2.228766	3.131327
H	1.514341	-2.025950	3.261187
H	-2.379640	0.041679	3.450576

Atom	x	y	z
H	-0.937330	3.843468	4.007613
H	-0.905890	-3.277050	4.473647
H	4.110619	-0.688760	3.947251
H	4.149560	0.830868	3.903036
H	-2.273750	3.126572	4.134346

Table S8. Cartesian structural coordinates of optimized structure B-β-1.

Atom	x	y	z
W	-0.481190	0.567564	-3.498980
W	1.189080	2.978407	-1.723940
W	2.598152	-0.223200	-2.086780
W	2.824324	-0.514230	1.654663
W	-1.811550	-2.652010	1.648801
W	-2.066650	-2.386250	-1.781960
W	1.031422	-3.124550	-0.333090
W	1.426107	2.686463	1.983654
W	-0.031290	0.014502	3.610633
Ni	-2.105040	2.229832	1.771253
Ni	-3.541680	0.012283	0.240949
Ni	-2.318950	2.430746	-1.198700
P	-0.319460	0.220040	0.010895
O	-0.644470	-1.291380	-0.067200
O	0.554967	0.495607	1.259347
O	0.422992	0.668115	-1.273060
O	-1.664700	1.030104	0.110572
O	2.932726	1.323836	1.957630
O	4.371366	-1.070220	2.142942
O	1.786908	-0.755370	3.195014
O	-2.682890	3.494686	0.312234
O	-3.850970	1.158645	1.680539
O	1.789296	2.893595	0.109707
O	-0.733290	-1.626220	2.845438
O	-2.952800	-3.346650	-2.894680
O	2.672429	1.643744	-2.130220
O	-0.464040	3.385588	-1.220800
O	0.965696	1.662530	3.576434
O	0.491851	2.223092	-3.378290

Atom	x	y	z
O	-0.198290	-3.594750	1.066517
O	-2.559630	-3.781260	2.702493
O	-1.136720	-1.167000	-2.923270
O	2.008328	-2.145190	0.962595
O	4.055895	-0.681040	-2.865370
O	-2.462110	3.447319	3.478558
O	2.217878	4.052214	2.664675
O	-4.069110	1.328297	-0.950350
O	-1.920040	1.374695	-2.715910
O	-3.263510	-1.081030	-1.269450
O	-2.454490	-3.242370	-0.087930
O	-0.149300	-0.298840	5.296308
O	-5.428310	-1.028800	0.331793
O	1.945209	-4.564270	-0.505510
O	-0.286730	3.190464	1.829070
O	3.078681	-0.177310	-0.237910
O	-0.389120	-3.389520	-1.590490
O	-3.039030	-1.280980	1.512427
O	1.841079	-1.952960	-1.589950
O	-1.540660	0.904931	3.217059
O	1.334905	-0.220170	-3.511050
O	1.872277	4.461500	-2.267600
O	-3.036100	3.666167	-2.311330
O	-0.881380	0.533879	-5.168920
H	-1.934390	4.109063	0.255001
H	-1.617920	3.917158	3.369846
H	-2.806100	3.272204	-3.166390
H	-5.244650	-1.525290	-0.482700
H	-2.233930	2.718041	4.080437
H	-3.679900	0.547889	2.417147
H	-4.024900	0.834812	-1.786440
H	-5.107470	-1.620930	1.032283

Table S9. Cartesian structural coordinates of optimized structure B-β2-1.

Atom	x	y	z
W	2.719071	2.402689	0.423784
W	3.476649	-0.994960	0.371482

Atom	x	y	z
W	2.175093	0.607388	-2.471040
W	-0.612350	-1.973500	-2.571800
W	-2.967640	0.713877	-1.574900
W	-2.366610	2.479690	1.395577
W	-0.419590	3.042839	-1.453010
W	0.766930	-3.514480	0.267027
W	-2.579580	-2.527530	0.189783
Ni	1.151597	-1.355460	3.086771
Ni	-1.787100	-0.498250	3.038956
Ni	0.417881	1.553737	3.208232
P	0.026885	0.016293	0.395291
O	-0.966710	1.100226	-0.092230
O	-0.395160	-1.381620	-0.128790
O	1.464668	0.336647	-0.084200
O	-0.002290	0.000539	1.972794
O	0.397022	-3.294600	-1.710260
O	-0.791950	-2.564210	-4.171620
O	-2.129780	-2.741040	-1.703150
O	1.574601	0.353151	4.086892
O	-0.705930	-1.763130	3.886344
O	2.338018	-2.483990	-0.101140
O	-3.245090	-0.828300	-0.473320
O	-3.462330	3.612244	2.086622
O	3.346795	-0.574370	-1.621130
O	2.899619	-1.104470	2.065451
O	-1.161360	-3.800380	0.411481
O	3.913222	0.883168	0.558787
O	-1.989570	2.229819	-2.243520
O	-4.357330	0.719882	-2.576790
O	1.099829	3.198779	-0.304900
O	-1.719620	-0.377670	-2.537210
O	2.813039	0.786137	-4.054450
O	2.357494	-2.723200	4.193845
O	1.352787	-5.129190	0.269866
O	-1.167590	0.728538	4.268426
O	1.990061	2.163344	2.022828
O	-0.841910	2.693430	2.378280

Atom	x	y	z
O	-3.477670	1.812925	-0.133900
O	-3.991090	-3.496470	0.306843
O	-3.667190	-0.877520	4.028659
O	-0.296930	4.468279	-2.395950
O	0.842929	-3.033300	1.995689
O	0.892030	-0.829030	-2.729730
O	-1.559250	3.592260	-0.040740
O	-2.789310	0.889890	2.259203
O	0.607676	1.773770	-2.441010
O	-2.405490	-1.836430	1.873441
O	2.986211	2.011640	-1.548120
O	5.035482	-1.715630	0.418448
O	0.716454	2.865786	4.420492
O	3.776419	3.745235	0.628375
H	2.438358	0.590895	3.717970
H	3.159227	-2.429320	3.728388
H	0.219339	3.584721	4.001050
H	-4.045100	-0.128790	3.532623
H	2.041325	-3.453710	3.632983
H	-0.894390	-2.574970	3.388665
H	-1.810140	1.451957	4.238872
H	-3.871490	-1.640630	3.463908

Table S10. Cartesian structural coordinates of optimized structure C-α-1.

Atom	x	y	z
W	2.909595	-2.129090	0.047136
W	0.148855	-2.894630	-1.926150
W	-2.946650	-0.776490	-1.646080
W	0.031396	3.306683	-1.010720
W	2.918559	1.349762	-1.270590
W	0.135871	0.617899	-3.254690
W	-2.919090	-1.413770	1.815720
W	-3.008900	1.880573	0.598201
Ni	0.006960	-0.436200	3.487026
Ni	0.004962	2.392404	2.369390
P	-0.002390	-0.038060	0.292728
O	0.518216	0.899147	-0.815830

Atom	x	y	z
O	-1.548460	-0.084290	0.295536
O	0.543209	-1.467640	0.050426
O	0.495830	0.472881	1.676272
O	-3.606480	-1.582990	-0.106800
O	-4.115920	-1.192370	-2.829890
O	-3.579390	0.906214	-1.023120
O	1.991649	-0.177740	3.793787
O	-0.431330	1.507549	3.950119
O	-1.740640	-2.771410	1.467834
O	-1.742890	2.737872	-0.636530
O	4.472345	1.837968	-1.811810
O	-0.176940	-3.927260	-0.422420
O	0.456957	-2.341940	2.913596
O	-3.721740	0.432450	1.548859
O	1.955324	-3.368990	1.084299
O	-0.173830	2.442759	-2.775820
O	-0.148730	4.962908	-1.429350
O	3.108365	-0.539930	-1.074340
O	-1.638810	0.223252	-2.695490
O	0.034453	-4.042520	-3.194940
O	-0.543180	-1.476550	5.279890
O	-4.224490	-2.205770	2.615618
O	1.829925	2.366435	2.817362
O	3.044489	-0.920020	1.475914
O	3.027841	1.459475	0.569857
O	1.932170	3.008737	-1.336030
O	-4.249590	3.059954	0.732572
O	-0.449450	4.375928	3.091931
O	0.012562	0.656710	-4.965330
O	-1.960770	-0.704110	3.171291
O	-1.641240	-2.181210	-1.832430
O	1.996017	0.963016	-2.963500
O	0.351078	3.358505	0.795835
O	0.479294	-1.222920	-2.887060
O	-1.825740	2.359849	1.941614
O	2.025775	-2.901870	-1.524780
O	0.121167	-5.170270	1.907072

Atom	x	y	z
O	4.323326	1.141124	3.115780
O	4.467006	-2.844470	-0.026660
H	-0.251410	-2.308250	4.864529
H	-0.125890	4.734391	2.246425
H	-1.468480	-1.406670	4.978418
H	-1.397980	1.462424	3.855551
H	-1.398050	4.249698	2.922551
W	2.757324	0.796170	2.487340
Ni	0.093719	-3.105590	1.259564
H	1.428533	-2.331460	2.922201
H	1.092523	-5.160810	1.851743
H	-0.135800	-5.419320	1.001363

Table S11. Cartesian structural coordinates of optimized structure D-α-1.

Atom	x	y	z
W	3.329033	-0.042100	-0.974050
W	1.074043	-2.424290	-2.130890
W	-2.418690	-2.494650	-0.727240
W	-2.301370	2.597627	-0.570600
W	0.940588	2.670662	-1.883650
W	-1.350460	0.284867	-3.018400
W	-3.305390	-0.164890	1.704075
Ni	1.221848	-0.045390	3.291156
Ni	-0.758520	2.154920	2.548147
P	0.000647	-0.009230	0.319194
O	-0.471160	0.952494	-0.790540
O	-1.192340	-0.911640	0.731450
O	1.158373	-0.909900	-0.163690
O	0.491488	0.817677	1.545672
O	-1.969550	-3.414550	0.814123
O	-3.408810	-3.626050	-1.552570
O	-3.679930	-1.442770	0.219871
O	2.615386	1.421885	3.078439
O	-0.019810	1.389988	4.087600
O	0.574335	-3.153670	1.643277
O	-3.230010	1.145463	0.246914
O	1.677563	3.918224	-2.804770

Atom	x	y	z
O	1.904329	-3.309730	-0.723800
O	2.423708	-1.433900	2.505868
O	-2.722420	-1.673070	2.671434
O	3.622298	-1.517630	0.135044
O	-2.495540	1.619437	-2.272120
O	-3.513840	3.802783	-0.736800
O	2.223677	1.265427	-1.935310
O	-2.298380	-1.024710	-2.018310
O	1.270260	-3.513930	-3.441370
O	1.844512	-1.041410	5.083517
O	-1.768660	-4.065740	3.394770
O	0.742672	3.256574	2.422181
O	3.163780	1.159444	0.462807
O	1.502390	2.996403	-0.152520
O	-0.790010	3.433337	-1.468940
O	-4.878280	0.077418	2.351397
O	-2.044690	3.514144	3.612051
O	-1.977530	0.091723	-4.603650
O	-0.297590	-1.401290	3.504718
O	-0.647230	-2.881190	-1.444370
O	-0.055260	1.680083	-3.257630
O	-1.556330	2.970731	1.062062
O	0.093832	-0.954930	-2.959760
O	-2.247850	0.996510	2.671140
O	2.632952	-1.308170	-2.287680
O	3.260989	-3.942420	1.703748
O	3.440563	3.733562	1.826164
O	4.895761	0.285737	-1.589970
H	2.490125	-1.538960	4.549710
H	-2.310950	3.924355	2.771024
H	1.041369	-1.590040	5.003121
H	0.609212	2.068397	4.376864
H	-2.706930	2.811971	3.730002
W	2.271285	2.479967	1.670254
W	2.267592	-2.634230	1.179414
Ni	-1.076110	-2.426000	2.154192
H	-1.053250	-0.859950	3.788517

Atom	x	y	z
H	-2.057800	-4.520220	2.584257
H	-2.542930	-3.514440	3.608164

Table S12. Cartesian structural coordinates of optimized structure B-α-2.

Atom	x	y	z
W	-3.147750	0.356200	1.740788
W	-3.147750	0.356200	-1.740790
W	-2.167960	-2.513490	0.000000
W	1.078185	-2.519600	-1.873830
W	3.081434	0.349414	1.857217
W	0.067742	0.350243	3.597542
W	1.078185	-2.519600	1.873832
W	0.067742	0.350243	-3.597540
W	3.081434	0.349414	-1.857220
Ni	-0.858290	3.167386	-1.495850
Ni	1.729888	3.165398	0.000000
Ni	-0.858290	3.167386	1.495852
P	0.000573	0.410788	0.000000
O	0.734057	-0.109470	1.270643
O	0.734057	-0.109470	-1.270640
O	-1.467970	-0.106070	0.000000
O	0.002652	1.968124	0.000000
O	0.427777	-1.622640	-3.390520
O	1.432251	-4.085260	-2.494040
O	2.718971	-1.624040	-2.058570
O	-1.723050	4.175461	0.000000
O	0.866000	4.181709	-1.489780
O	-1.693670	-0.075210	-2.935700
O	3.396538	-0.069620	0.000000
O	-0.166490	0.407547	5.303710
O	-3.154870	-1.615470	-1.323440
O	-2.598420	2.064122	-1.682560
O	1.997834	0.464041	-3.459220
O	-3.993490	0.482304	0.000000
O	2.718971	-1.624040	2.058570
O	4.674867	0.404063	2.510303
O	-1.693670	-0.075210	2.935696

Atom	x	y	z
O	1.467389	-2.728670	0.000000
O	-2.881430	-4.079560	0.000000
O	-1.878000	4.079153	-3.276780
O	-0.166490	0.407547	-5.303710
O	0.866000	4.181709	1.489777
O	-2.598420	2.064122	1.682561
O	-0.143000	2.059824	3.093139
O	1.997834	0.464041	3.459222
O	4.674867	0.404063	-2.510300
O	3.786384	4.070726	0.000000
O	1.432251	-4.085260	2.494040
O	-0.143000	2.059824	-3.093140
O	-0.744060	-2.710380	-1.279420
O	0.427777	-1.622640	3.390522
O	2.752969	2.059609	1.421450
O	-0.744060	-2.710380	1.279422
O	2.752969	2.059609	-1.421450
O	-3.154870	-1.615470	1.323436
O	-4.506960	0.413588	-2.797800
O	-1.878000	4.079153	3.276778
O	-4.506960	0.413588	2.797802
H	-2.598490	3.766897	0.000000
H	1.303786	3.774743	2.248988
H	1.303786	3.774743	-2.248990
H	3.881392	3.459143	0.755450
H	-1.272510	3.466123	-3.736690
H	-2.582730	3.469138	2.985086
H	-1.272510	3.466123	3.736685
H	3.881392	3.459143	-0.755450
H	-2.582730	3.469138	-2.985090

Table S13. Cartesian structural coordinates of optimized structure A-α-2.

Atom	x	y	z
W	1.010721	2.626912	1.746872
W	-2.013369	2.626834	0.000000
W	1.010721	2.626912	-1.746872
W	3.174434	-0.452014	1.727539

Atom	x	y	z
W	-0.094979	-0.446129	3.615556
W	-3.084212	-0.449102	1.886666
W	-3.084212	-0.449102	-1.886666
W	-0.094979	-0.446129	-3.615556
W	3.174434	-0.452014	-1.727539
Ni	1.963805	-2.950273	0.000000
Ni	-0.982297	-2.945922	-1.704498
Ni	-0.982297	-2.945922	1.704498
P	0.000259	-0.368297	0.000000
O	1.623811	3.843036	2.801471
O	-3.237358	3.838865	0.000000
O	0.003993	1.179668	0.000000
O	1.623811	3.843036	-2.801471
O	3.307335	-2.227231	1.452823
O	4.496732	-0.140562	2.783961
O	-0.397219	-2.222071	3.593722
O	0.158562	-0.133191	5.288595
O	-2.909348	-2.223571	2.142921
O	-2.909348	-2.223571	-2.142921
O	-0.397219	-2.222071	-3.593722
O	0.158562	-0.133191	-5.288595
O	3.307335	-2.227231	-1.452823
O	4.496732	-0.140562	-2.783961
O	-0.734844	-0.887331	1.272789
O	-0.734844	-0.887331	-1.272789
O	1.468611	-0.892275	0.000000
O	0.849730	-3.644830	-1.473857
O	-1.703769	-3.633772	0.000000
O	0.849730	-3.644830	1.473857
O	-0.754120	3.320406	1.308081
O	1.509248	3.322231	0.000000
O	2.422694	1.407193	1.609751
O	0.184406	1.411087	2.906751
O	-0.754120	3.320406	-1.308081
O	-2.600373	1.402713	1.289942
O	-2.600373	1.402713	-1.289942
O	0.184406	1.411087	-2.906751

Atom	x	y	z
O	2.422694	1.407193	-1.609751
O	1.696707	-0.672156	2.941679
O	3.945920	-0.058889	0.000000
O	-1.976056	-0.054143	3.421668
O	-3.402178	-0.676835	0.000000
O	-1.976056	-0.054143	-3.421668
O	1.696707	-0.672156	-2.941679
O	-4.659911	-0.136763	2.504176
O	-4.659911	-0.136763	-2.504176
O	3.676527	-4.451266	0.000000
O	-1.832146	-4.448871	3.190658
O	-1.832146	-4.448871	-3.190658
H	-2.609750	-3.299278	0.000000
H	1.306246	-3.315378	2.258641
H	1.306246	-3.315378	-2.258641
H	-2.609775	-3.879309	-3.029097
H	3.921115	-3.878624	-0.753577
H	-1.301970	-3.874011	3.777385
H	-2.609775	-3.879309	3.029097
H	-1.301970	-3.874011	-3.777385
H	3.921115	-3.878624	0.753577

Table S14. EXAFS Fitting Parameters for Cs_4K-1.

Path	Fitted C.N.	R (Å)	$\sigma^2/10^{-3}$
Ni-O	5.40 ± 0.74	2.00 ± 0.01	6.4 ± 1.7
Ni-Ni	2.00 (Fixed)	3.13 ± 0.06	9.8 ± 5.5
Ni-P	1.00 (Fixed)	3.17 ± 0.30	9.8 ± 5.5
Ni-W	2.00 (Fixed)	3.58 ± 0.13	9.8 ± 5.5

S_0^2 fixed at 0.935 $\Delta E_0(O) = -7.72 \pm 1.09$ R factor = 2.63% Fit k^2 with bg

ΔE_0 (Ni) = 0.35 ± 8.45

ΔE_0 (P) = -4.55 ± 19.97

ΔE_0 (W) = -12.07 ± 19.10

Table S15. EXAFS Fitting Parameters for Cs_4KH-2.

Path	Fitted C.N.	R (Å)	$\sigma^2/10^{-3}$
Ni-O	5.71 ± 0.76	2.03 ± 0.01	7.3 ± 1.8
Ni-Ni	2.00 (Fixed)	3.13 ± 0.18	13.3 ± 11.1
Ni-P	1.00 (Fixed)	3.44 ± 0.62	13.3 ± 11.1
Ni-W	2.00 (Fixed)	3.55 ± 0.09	7.0 ± 3.6

S_0^2 fixed at 0.935	ΔE_0(O) = -3.80 ± 2.01<br>ΔE_0 (Ni) = 1.41 ± 31.24<br>ΔE_0 (P) = 3.67 ± 17.58<br>ΔE_0 (W) = -14.09 ± 14.27	R factor = 2.02%	Fit k^2 with bg

Table S16. EXAFS Fitting Parameters for PW_9Ni_3/MIL-101(Cr) composite.

Path	Fitted C.N.	R (Å)	$\sigma^2/10^{-3}$
Ni-O	6.30 ± 1.03	2.03 ± 0.01	7.0 ± 2.0
Ni-Ni	2.00 (Fixed)	3.15 ± 0.09	8.5 ± 4.5
Ni-P	1.00 (Fixed)	3.14 ± 0.29	8.5 ± 4.5
Ni-W	2.00 (Fixed)	3.61 ± 0.13	8.5 ± 4.5

S_0^2 fixed at 0.935	ΔE_0(O) = -4.64 ± 1.67<br>ΔE_0 (Ni) = 1.51 ± 9.34<br>ΔE_0 (P) = 6.73 ± 20.44<br>ΔE_0 (W) = -10.29 ± 18.57	R factor = 2.23%	Fit k^2 with bg

Table S17. Calculated pore volume and surface area of MIL-101(Cr) and the composite.

Compound	Specific surface area (m^2/g)	Pore volume (cm^3/g)
MIL-101(Cr)	2010	1.18
PW_9Ni_3/MIL-101(Cr)	1365	0.74

Table S18. Comparison of onset overpotentials of different POM-based WOCs under neutral pH.

Compound	Onset overpotential (V)	Reference
$K_2[Cr_3O(O_2CCH_2CN)_6(H_2O)_3]_4$ $[\alpha\text{-}Co^{II}W_{12}O_{40}]\cdot 32H_2O$	0.66	1
$[\alpha\text{-}Co^{II}W_{12}O_{40}]^{6-}$	0.90	1
$[Mn^{II}Mn^{III}SiW_{10}O_{37}(OH)(H_2O)]^{6-}$	0.66	2
$[Mn^{II}_3Mn^{III}(H_2O)_2(PW_9O_{34})_2]^{9-}$	0.64	2
$[Mn^{II}_4Mn^{III}_2Ge_3W_{24}O_{94}(H_2O)_2]^{18-}$	0.60	2
$[Mn^{II}_{19}(OH)_{12}(SiW_{10}O_{37})_6]^{34-}$	0.42	2
$\{Fe_{10}P_4W_{32}\}^a$	≥0.68	3
$[Co_9(H_2O)_6(OH)_3(HPO_4)_2(PW_9O_{34})_30]^{16-}$	0.48	4
**Cs_4K-1**	0.64	This work
PW_9Ni_3/MIL-101(Cr) composite	0.64	This work

[a] Formulated as $[Na(H_2O)Fe^{II}(H_2O)_2(DAPSC)]_2\{[Fe^{II}(H_2O)(DAPSC)]_2[Fe^{II}(H_2O)_4]_2$ $[Na_2Fe^{III}_4P_4W_{32}O_{120}]\}\cdot 25H_2O$, where DAPSC represents 2,6-diacetylpyridine bis(semicarbazone).

References

1. Yuto Shimoyama, Naoki Ogiwara, Z. Weng and S. Uchida, *Journal of the American Chemical Society*, 2022, **144**, 2980–2986.
2. Y. Wu, J. Pei, X. Yu and L. Bi, *Catalysts*, 2022, **12**, 160.
3. W. Jiang, X.-M. Liu, J. Liu, J. Shi, J.-P. Cao, X.-M. Luo, W.-S. You and Y. Xu, *Chemical Communications*, 2019, **55**, 9299–9302.
4. M. Blasco-Ahicart, J. Soriano-López, J. J. Carbó, J. M. Poblet and J. R. Galan-Mascaros, *Nature Chemistry*, 2017, **10**, 24–30.
